# Anti-ageing and attenuating cognitive declines of *Dicliptera chinensis* extracts and purified compounds in vitro and in scopolamine-induced cognitive dysfunction mice

**DOI:** 10.1186/s40529-025-00478-8

**Published:** 2025-09-29

**Authors:** Yi-Yan Sie, Mei-Hsien Lee, Wen-Chi Hou

**Affiliations:** 1 Ph.D. Program in Clinical Drug Development of Herbal Medicine, College of Pharmacy, Taipei Medical University, Taipei, 110 Taiwan; 2 Graduate Institute of Pharmacognosy, Taipei Medical University, Taipei, 110 Taiwan

**Keywords:** Acetylcholinesterase (AChE), Anti-ageing, *Dicliptera chinensis* (L.) Nees (DC), Reactive oxygen species (ROS), Scopolamine, Senescence-associated β-galactosidase (SAβG) activity

## Abstract

**Background:**

The cognitive decline is one of the age-associated physical dysfunctions and also found in Alzheimer’s disease (AD). The whole plants of *Dicliptera chinensis* (L.) Nees (DC) are belonged to edible materials as fresh vegetables, and dried DC powders as tea materials or used as folk medicines for clearing heat and removing toxic substance in Taiwan. However, DC extracts and the active compounds on anti-ageing and attenuating cognitive declines are not clear.

**Results:**

The dried powders of the whole DC plants are extracted with 95% ethanol to get DC-95EE. The DC-95EE is partitioned in sequences to get ethyl acetate fraction, butanol fraction (BuOH fraction), and water faction. Two flavone C-glycosides (vicenin II and schaftoside) are purified from the BuOH fraction. It is found that the pre-treatments of DC-95EE, BuOH fraction, and vicenin II and schaftoside show to elevate cell viabilities, reduce the senescence-associated β-galactosidase (SAβG) activity and intracellular reactive oxygen species (ROS) levels in galactose-treated SH-SY5Y neuronal cell models. The pre-treatments of vicenin II or schaftoside also show to lower ageing-associated p16 and p21 gene expressions and enhance SIRT-1 gene expressions in galactose-treated SH-SY5Y cells. The DC-95EE, vicenin II, and schaftoside show dose-dependent anti-acetylcholinesterase activities. The oral administrations of DC-95EE (200 mg/kg) and purified schaftoside (25 and 50 mg/kg) daily for 7 days ameliorate cognitive declines in scopolamine-induced ICR mice evaluated by Morris water maze. The quantification of vicenin II and schaftoside, respectively, account for 2.53% and 8.17% of DC-95EE by HPLC quantifications, which may be active compounds in the DC-95EE for anti-ageing activities.

**Conclusion:**

The DC-95EE exhibit anti-ageing in neuron cell models and attenuate cognitive declines in mice models, which may be potentials in developing functional foods for anti-ageing purposes and need further investigations.

**Supplementary Information:**

The online version contains supplementary material available at 10.1186/s40529-025-00478-8.

## Background

The progresses in public health and medical treatments contribute population ageing in many countries. The aged person older than 60-year-old in 2019 globally is one billion, and is estimated to increase to 1.4 and 2.1 billion by 2030 and 2050, respectively (World Health Organization 2025). The countries in Asia and the Pacific regions, the numbers of the aged people older than 60 years are estimated from 630 million of about 13.6% of total population in 2020 to more than double of 1.3 billion by 2050 of about 25% of total population (Economic and Social Commission for Asia and the Pacific 2025). The World Health Organization used “Healthy ageing” to define wellbeing of the aged people during ageing process by developing and maintaining the functional abilities (World Health Organization 2020). These functional abilities were defined by self-capabilities of aged people to encounter disease, injury, and age-associated physical dysfunctions (such as memory, vision, walking, hearing, or thinking), or social supports and related public health policies. Based on the statistic information of the National Institute on Aging, the greatest risk factor of Alzheimer’s disease (AD) is the older age, the other AD-associated factors include genetics (APOE ɛ4 gene) and the family history etc. (National Institute on Aging 2025). At present, no long-term effective drug can delay cognitive declines in AD progressions, and the unmet needs on medicines or functional foods are currently developing in AD treatments, which the targets are mainly focused on the pathological theories of AD, including amyloid-β (Aβ) peptide aggregations to generate amyloid-like fibrils, hyper-phosphorylated Tau protein aggregations to form neurofibrillary tangles, and low levels of acetylcholine and neuron deaths (Querfurth and LaFerla 2010; Craig et al. 2011; Selkoe and Hardy 2016). The developing nutraceuticals or functional foods are mainly focused on natural products to provide health benefits to protect and encounter diseases from foods, vegetables, fruits, folk herbs, and traditional Chinese medicines with the same source of foods. The major protein of yam tuber, dioscorins, has been reported to exhibitanti-aging in galactose-induced oxidative stress mice models (Han et al. 2014a, b). The dry powders of small-leaf-grape (*Vitis thunbergii* var. *taiwaniana*), an endemic herbal plant of Taiwan rich in resveratrol and resveratrol oligomers, or the water caltrop (*Trapa taiwanesis* Nakai) hull powders, rich in hydrolysable tannins, are materials used as substituents for tea leaves, which both extracts are reported to exhibit blood glucose regulation in type 2 diabetic animal models (Yasuda et al. 2014; Liu et al. 2023) and also have improved learning dysfunctions in scopolamine-induced mice models (Chen et al. 2021, 2022). The reactive oxygen species (ROS) in cells are generated either by mitochondrial electron transport chains for energy productions, metabolic enzymes of xanthine oxidase, amine oxidase and NAD(P)H oxidase, and environmental factors (such as UV radiation, chemicals, or pathogen attacks), which can be scavenged by non-enzymatic compounds or metabolized by antioxidant enzyme systems. The advanced glycation end-products (AGEs) are formed by non-enzymatic glycations reactions in cells among carbonyl groups in reducing sugars and amino groups in proteins (Zhang et al. 2009), and these AGEs can bind onto AGE receptors to increase ROS productions through activation of NADPH oxidase (Calcutt et al. 2009). The situations of the ROS productions are higher than those be scavenged as described by the theory of “free radical theory of aging” (Cui et al. 2012). Cells under the oxidative stress and accumulated oxidative damages are shifted to decline the proliferation and differentiation abilities as well as the physiological functions, which are the status of the senescent cells including cell-cycle arrests, senescence-associated secretory phenotypes, and the metabolic dysfunctions and macromolecular damages, and are in progress toward cell deaths (Zhu et al. 2021; Guo et al. 2022). Hesperidin shows the anti-senescent activities in doxorubicin-induced lung fibroblast via reductions of protein expressions of p53, p21, p16 and numbers of senescence-associated β-galactosidase (SAβG)-positive cells, and also have abilities to suppress bleomycin-induced pulmonary fibrosis rat models (Han et al. 2023). The extracts of smoked cigarettes contain pro-senescent factors which can induce alveolar epithelial type 2 cell senescence to pulmonary fibrosis via inactivation of SIRT-1 (Zhang et al. 2021). Urolithin A shows to attenuate cell senescence by lowering gene expressions of senescence-associated p53 and p21 and reductions of SAβG-positive cells in hydrogen peroxide-treated auditory cells (Cho et al. 2022). Nobiletin shows to prevent D-galactose-induced C2C12 cell ageing by elevating the antioxidant capacities, anti-inflammatory activities, and mitochondrial functions to reduce intracellular ROS, numbers of SAβG-positive cells, and senescence-associated p53, p21, and p16 protein expressions (Wang et al. 2022).

The *Dicliptera chinensis* (L.) Nees (DC), also called Chinese foldwing, a perennial double folding wing herbal plant in the family Acanthaceae mainly distributed in tropical and subtropical regions of Vietnam, northeast India and southern China, are edible fresh vegetables. The dried powders of DC plants as tea materials in Taiwan or used as folk medicines in clearing heat in common cold and removing toxic substance and detoxification, liver disease treatments and diuretic actions (Zhang et al. 2010). Some small molecular compounds were identified in the extracts of *Dicliptera* species. Luo et al. (2002) reported that vanillic acid, β-sitosterol, lugrandoside, 2,5-dimethoxy-*p*-benzoquinone, poliumonside daucosterol, dicliripariside B and dicliripariside C (flavonoid glycosides), and dicliripariside A (a dimeric monoterpenoid glucoside) were isolated and purified from the ethanolic extracts of the whole plants of *D. riparia*. Gao et al. (2006) reported that secoisolariciresinol dimethyl ether diacetate, chinensinaphthol methyl ester, β-sitosterol 3-*O*-β-d-glucopyranoside, stigmasterol 3-*O*-β-d-glucopyranoside, loliolide, and 5-methoxy-4,4′-di-*O*-methyl secolariciresinol were isolated and purified from the ethyl acetate fractions of DC ethanolic extracts. Gao et al. (2007a, b) reported that 3β-hydroxy-stigmast-5-en-7-one, 6β-hydroxy-stigmast-4-en-3-one, stigmast-5-en-7-oxo-3β-yl palmitate, stearic acid, lupenone, dicliptercerebroside, amidoalcohol, asperglaucide, hexatriacontanol, oleanolic acid, lupeol, vomifoliol, dehydrovomifoliol, 4-sitost-4-en-3-one, and 3β,6β-stigmast-4-en-3,6-diol were purified from DC whole plants. Huang et al. (2012) reported that gardenoside, kaempferitrin, chlorogenic acid, ferulic acid, epicatechin, and daucosterol were isolated and purified from the ethyl acetate fractions of DC ethanolic extracts. Zou et al. (2012) reported that diosgenin, 1-octadecanol, oleanic acid, β-stitosterol, ursolic acid, isoalantolactone, scoparone, and 7-hydroxycoumarin were isolated and purified from the chloroform fractions of DC ethanolic extracts. The boiling water-extracted polysaccharides, with molecular mass of 2273 Da, from dried DC plants have shown free radical scavenging activities in vitro against DPPH, hydroxyl radicals, ABTS radicals and superoxide radicals, and oral administrations of the DC polysaccharides have ameliorated galactose-induced ageing mice by reducing oxidative stress marker of the malondialdehyde (MDA) levels, increasing total antioxidant capacities and antioxidant enzyme activities of glutathione peroxidase and superoxide dismutase (Xu et al. 2017). The DC polysaccharides have proved to exhibit hepatoprotective activities against dimethylnitrosamine-induced rat hepatic fibrosis via improvements of inflammation and necrosis, reductions liver functions of alanine aminotransferase, aspartate aminotransferase, and total bilirubin in serum levels (Zhang et al. 2016). The kaempferol-3-*O*-β-d-glucopyranoside, nicotiflorin, kaempferol-3-*O*-α-l-rhamnopyranoside-7-β-d-glucopyranoside, catechin, and quercetin isolated from ethyl acetate (EA) fractions of ethanol extracts of DC leaves have shown anti-inflammatory activities via dose-dependent cyclooxygenase-2 inhibitory activities in vitro (Duc et al. 2018). The isolated β-sitosterol from chloroform fractions and vanillic acid from EA fractions of methanol extracts of the dried DC plants have exhibited antibacterial activities against *Penicillium notatum*, which show better activities than the anti-fungi drug of fluconazole, and also exhibit DPPH radical scavenging activities and cyto-toxicities against Hep G2 cell lines (Akbar et al. 2021). DC extracts and/or purified compounds have been reported to exhibit several biological activities, however, the anti-ageing and attenuating cognitive declines by using DC extracts were not clear. Therefore, in this study, DC extracts, partitioned fractions, and purified compounds are used to investigate anti-ageing activities in galactose-treated SH-SY5Y cell models, and DC extracts and purified compound are used in the prevention mode to treat scopolamine-induced cognitive dysfunction ICR mice for evaluating improvements of learning behaviors.

## Methods

### Materials

The recombinant human acetylcholinesterase (rhAChE) was purchased from R&D Systems Inc. (Minneapolis, MN). The senescence detection kit (K320-250) was from BioVision Inc. (San Francisco, CA). The Aβ_1-42_ peptide, thioflavin T (ThT), acetylthiocholine iodide, 3-[(3-cholamidopropyl)dimethylammonio]-1-propanesulfonate (CHAPS), 5,5′-dithiobis(2-nitrobenzoic acid) [DTNB], 3-(4,5-dimethyl-2-thiazolyl)-2,5-diphenyl-2H-tetrazolium bromide (MTT, 475989), 4-methylumbelliferyl-β-d-galactopyranoside (4-MUG), 5-bromo-4-chloro-3-indolyl-β-d-galactopyranoside (X-gal), D-galactose, 2′,7′-dichlorofluorescin diacetate (DCFH-DA), dimethyl sulfoxide (DMSO), phosphate buffered saline (PBS), 1,1,1,3,3,3-hexafluoroisopropanol (HFIP), 0.01% poly-l-lysine solution, polyethylene glycol (PEG) 400, phenylmethanesulfonyl fluoride (PMSF), and scopolamine hydrobromide were purchased from Sigma Chemical Co. (St. Louis, MO). The ellagic acid was purchased from Fluka Chemie GmbH (Buchs, Switzerland). The vicenin II and schaftoside with purity higher than 99.5% were purchased MedChemExpress LLC (NJ, USA). TLC aluminum sheets silica gel 60 F254 (20 × 20 cm) was from Merck Co. (Darmstadt, Germany). The donepezil hydrochloride (D4099) was from Tokyo Chemical Industry Co. (Tokyo, Japan). The BCA Protein Assay Kit (Catalog No. PI23225) was purchased from ThermoFisher Scientific Inc. (Rockford, IL, USA). The fetal bovine serum (FBS), Dulbecco’s modified eagle medium (DMEM), and DMEM/F-12 medium were purchased from Gibco BRL Life Technologies (Grand Island, NY). ^1^H- and ^13^C-NMR spectra were measured at room temperature and reported in ppm by using the DMSO-*d*_*6*_ solvent resonance. The ^1^H-NMR (500 MHz) and ^13^C-NMR (125 MHz) spectra were measured at room temperature by Nuclear Magnetic Resonance Spectrometer (Bruker, Rheinstetten, Germany), and the molecular mass was measured by Time-of-Flight Secondary Ion Mass Spectrometer (ULVAC-PHI TRIFT IV, Kanagawa, Japan). The X-ray Single Crystal Diffractometer (Bruker D8 VENTURE, Rheinstetten, Germany) was used for crystal diffractions. The ICP-MS (Thermo X Series II, Thermo Fisher Scientific Inc, Massachusetts, USA) was used for elementary analyses.

### Preparations of crude extracts and partitioned fractions

The DC whole plants were purchased from a local herbal store in April 2019. The voucher specimens were kept in the Graduate Institute of Pharmacognosy, Taipei Medical University for references. The DC whole plants were air-dried at open space for 1-week, ground mechanically to fine powders, and then were extracted by soaking in 95% ethanol (W/V = 1:4) at room temperature (27 °C) for 24 h. After being filtered, the residues were repeated for another four times at the same procedure. Each 95% ethanol extracts were collected, combined and concentrated by a vacuum concentrator, and lyophilized to get DC-95EE (140.2 g) for further experiments. For preparations of DC-95EE partitioned fraction (fr.), the DC-95EE (132.7 g) was weighted and suspended in distilled water and then partitioned in sequence by *n*-hexane, ethyl acetate (EA), and *n*-butanol (BuOH), respectively, to get hexane fr. (54.1 g), EA fr. (3.8 g), BuOH fr. (19.3 g), and water fr. (55.5 g).

### Isolation and purification from the butanol fractions of DC-95EE

The BuOH fraction of DC-95EE was weighted (19.3 g), dissolved in distilled water, and loaded onto a Diaion HP-20 column (70 cm × 3 cm), chromatographed and eluted by batch types of solvents in sequences of distilled water, 20% increases from 20% methanol to 100% methanol, and 70% acetone to obtain 7 fractions of DCB 1-1 to DCB 1-7. The fraction DCB 1-4 was weighted (1.39 g) dissolved in distilled water, and loaded onto a Sephadex LH column (45 cm × 2 cm), chromatographed and eluted by batch types of solvents in sequences of distilled water, 10% increases from 10% methanol to 100% methanol to get DCB 1-4-1 (collection of tube 1-21), DCB 1-4-2 (collection of tube 22–70), and DCB 1-4-3 (collection of tube 71-109). The sub-fraction DCB 1-4-1 was re-chromatographed with Toyoperal HW-40(F) column (45 cm × 2 cm) and eluted by batch types of solvents in sequences of distilled water, 10% increases from 10% methanol to 100% methanol to get DCB 1-4-1-1 (collection of tube 1-6), DCB 1-4-1-2 (collection of tube 41-90), and DCB 1-4-1-3 (collection of tube 91-101). The sub-fraction DCB 1-4-1-1 was re-chromatographed with Biosil Aqu-ODS-W-5u column (70.4 mm × 14.5 mm) and eluted with 10% methanol to get DCB 1-4-1-1-1 fraction. The DCB 1-4-1-1-1 fraction was further purified by semi-preparative HPLC using a Waters Atlantis T3 semi-preparative column (5 μm, 10 mm i.d. × 250 mm) eluted with 38% methanol, 2 mL/min, to get DCB1-4–1-1-1-1 fraction. The DCB 1-4-1-1-1-1 was divided into two parts, one DCB 1-4-1-1-1-1a was further chromatographed on a Waters Atlantis T3 semi-preparative column (5 μm, 10 mm i.d. × 250 mm) eluted with 35% methanol, 2 mL/min, to get compound **1** (13.5 mg); the other DCB 1-4-1-1-1-1b was further chromatographed on a VP-Nucleodur C18 HTec semi-preparative column (5 μm, 10 mm i.d. × 250 mm) eluted with 38% methanol, 2 mL/min, to get compound **2** (10 mg). Both of the BuOH fr. and water fr. contained needle-like white crystal precipitations, which the repeated recrystallizations by a small amount of water/methanol to get compound** 3** (7.5 g). The purity of each compound was determined by HPLC and was shown to exceed 95%. All structures were determined by ^1^H-NMR and ^13^C-NMR and by comparison of those data with authentic compounds as follows.

- Apigenin-6,8-di-*C*-glucopyranoside (Vicenin II, compound** 1**) (Yoshizaki et al. 1987; Velozo et al. 2009):
  Yellow amorphous powder. ESI [M+H]^+^ 595.17; UV(MeOH) λ_max_(logε):334 nm (4.15), 273 nm (4.13). ^1^H-NMR (500 MHz, DMSO-d_6_), δ, 13.71 (1H, *br s*, 5-OH), 8.00 (2H, *br s*, H*-*2′, 6′), 6.93 (2H, *d*, *J* = 6.7 Hz, H-3′, 5′), 6.78 (1H, *s*, H-3), 4.77 (1H, m, H-1″), 4.77 (1H, *m*, H-1‴), 3.87 (1H, *m*, H-2‴), 3.74 (1H, *m*, H-6‴), 3.64 (2H, *m*, H-6″), 3.55 (1H, *m*, H-2″), 3.51 (1H, *m*, H-6‴), 3.37 (1H, *m*, H-4‴), 3.34 (1H, *m*, H-4″), 3.28 (1H, *m*, H-5‴), 3.27 (1H, *m*, H-3″), 3.27 (1H, *m*, H-3‴), 3.22 (1H, *m*, H-5″). ^13^C-NMR (125 MHz, DMSO-d_6_), δ, 182.0 (C-4), 163.7 (C-2), 161.2 (C-7), 161.2 (C-4′), 158.8 (C-5), 155.1 (C-9), 128.9 (C-2′, 6′), 121.6 (C-1′), 115.9 (C-3′, 5′), 107.6 (C-6), 105.3 (C-8), 103.2 (C-10), 102.5 (C-3), 81.9 (C-5″), 80.9 (C-5‴), 78.9 (C-3‴), 78.0 (C-3″), 74.1 (C-1″), 73.4 (C-1‴), 71.8 (C-2″), 71.0 (C-2‴), 70.6 (C-4‴), 69.2 (C-4″), 61.3 (C-6‴), 59.9 (C-6″).
- Apigenin 6-*C*-β-d-glucopyranosyl-8-*C*-α-l-arabinopyranoside (Schaftoside, compound** 2**) (Xie et al. 2003):
  Yellow amorphous powder. ESI [M+H]^+^ 565.15; UV(MeOH) λ_max_(logε):335 nm (4.19), 273 nm (4.17). ^1^H-NMR (500 MHz, DMSO-d_6_), δ, 13.81 (1H, *br s*, 5-OH), 8.10 (2H, *br s*, H*-*2′, 6′), 6.90 (2H, *d*, *J* = 8.8 Hz, H-3′, 5′), 6.79 (1H, *s*, H-3), 4.76 (1H, *m*, H-1‴), 4.69 (1H, *d*, *J* = 9.0 Hz, H-1″), 4.06 (1H, *m*, H-2‴), 3.87 (1H, *m,* H-2″), 3.87 (1H, *d*, *J* = 10.5 Hz, H-5‴), (1H, *m*, H-3″), 3.22 (1H, *m*, H-4″), 3.22 (1H, *m*, H-5″), 3.66 (1H, *d*, *J* = 11.4 Hz, H-6″), 3.48 (1H, *m*, H-6″), 3.48 (1H, *m*, H-3‴), 3.84 (1H, *d*, *J* = 10.5 Hz, H-4‴), 3.66 (1H, *d*, *J* = 11.4 Hz, H-5‴). ^13^C-NMR (125 MHz, DMSO-d_6_), δ, 182.2 (C-4), 163.8 (C-2), 162.2 (C-7), 161.1 (C-4′), 159.5 (C-5), 154.3 (C-9), 129.1 (C-2′, 6′), 121.6 (C-1′), 116.0 (C-3′, 5′), 108.5 (C-6), 104.5 (C-8), 103.1 (C-10), 102.3 (C-3), 81.4 (C-5″), 78.6 (C-3″), 75.5 (C-1″), 74.9 (C-1‴), 74.6 (C-3‴), 70.7 (C-2″), 70.7 (C-5‴), 70.0 (C-4″), 68.8 (C-2‴), 68.8 (C-4‴), 60.9 (C-6″).
- Potassium nitrate (compound** 3**) (Freney et al. 2009):
  The compound **3** was analyzed by ICP-MS, elementary analyses, and X-ray diffractions. The white crystal (orthorhombic crystal system) was proposed to be KNO_3_with formula weight of 101.11 Da.

### The HPLC fingerprints of DC-95EE and quantifications of compound 1 and 2 in the DC-95EE

For DC-95EE fingerprinting analyses, the analytical Galaksil EF-C18 (5 μm, 250 × 4.6 mm) was performed using Shimadzu SCL-40 chromatography system with a gradient elution program was set in solvent mixtures of distilled water and acetonitrile as follows: water/acetonitrile, 90/10, at 0–35 min; 0/100, at 35–40 min; 90/10, at 40.01–55 min. The concentrations of DC-95EE, compound **1**, and compound **2*,*** respectively, were 100 mg/mL, 1 mg/mL, and 1 mg/mL, and 10 μL was injected for analyses. The flow rate was 1.0 mL/min and the wavelength was set at 335 nm for monitoring. The identified compounds included compound **1** (vicenin II, 7.415 min) and compound **2** (schaftoside, 8.112 min). For quantifications of compound **1** and compound **2** in the DC-95EE, the analytical Phenomenex Luna PFP(2) (5 μm, 250 × 4.6 mm) was performed using Shimadzu SCL-40 chromatography system with an isocratic elution program was set in solvent mixtures of distilled water and acetonitrile as follows: water/acetonitrile containing 0.05% acetic acid, 87/13, at 0–30 min; 0/100, at 30.01–35 min; 87/13, at 35.01–40 min. For plotting standard curves of compound **1** and compound **2** of concentrations versus area in HPLC chromatograms, nine concentrations of the two-fold dilution from 500, 250, 125, 62.5, 31.25, 15.625, 7.8125, 3.90625–1.953 μg/mL were prepared. The prepared concentration of DC-95EE was 100 mg/mL and 10 μL was injected for analysis. The flow rate was 1.0 mL/min and the wavelength was set at 335 nm for monitoring. The identified compounds included compound **1** (vicenin II, 11.426 min) and compound **2** (schaftoside, 21.341 min).

### Neuroprotective and anti-ageing activities of DC-95EE, partitioned fractions and its purified compounds pre-treatments in galactose-treated SH-SY5Y neuronal cell models

The galactose-treated SH-SY5Y cells (the human neuroblastoma cell lines from American Type Culture Collection, Manassas, VA) were used as in vitro neuronal cell models to investigate neuroprotective and anti-ageing activities of DC-95EE, partitioned fractions and its purified compounds following the previous reports with modifications (Sie et al. 2023a, b). The SH-SY5Y cells (5 × 10^3^ cells/well) were seeded onto a 96-well microplate and cultured in a DMEM/F-12 medium containing 10% FBS for 24 h at 37 °C under a humidified atmosphere and 5% CO_2_. The culture medium was removed from the cultured plate, and the DC-95EE (50 μg/mL), the partitioned fractions (50 μg/mL), compound **1** and compound **2** (100 μM), and 0.1% DMSO (the control and the blank) were pre-treated and incubated at 37 °C in a humidified atmosphere with 5% CO_2_ for 24 h. The ellagic acid (5 μM) was used as the positive control. The treated media were removed, washed with PBS, and 200 mM of D-galactose in PBS were added to the medium for another 48-h culture. The equal aliquot of PBS was added in the blank. For cell viability, the MTT (0.5 mg/mM) was used and the absorbance at 600 nm was determined (Wang et al. 2006), and cell viability in the blank was recognized as 100%, and each treatment was expressed as relative cell viability (%).

For determinations of ROS levels, the DCFH-DA (20 μM) was added and incubated for 30 min after 200 mM D-galactose-treated cells, and the fluorescence (Ex_485nm_/Em_520nm_) was determined (Lin et al. 2020), and the ROS level in the blank was recognized as 100%, and each treatment was expressed as relative ROS levels (%). For SAβG activity determinations, the treated-cells were lysed by 5 mM CHAPS detergents in citric acid/sodium phosphate buffer (pH 6.0) containing protease inhibitors of 0.5 mM benzamidine and 0.25 mM PMSF. The cell lysates were divided into two parts, one for protein determinations by BCA protein assay kit, and the other was placed in the 96-well black microplate and the aliquots of 1.7 mM 4-MUG in reaction buffer [citric acid/sodium phosphate buffer (pH 6.0), 300 mM NaCl, 4 mM MgCl_2_, and 10 mM 2-mercaptoenthanol] were added and reacted in the incubator at 37 °C for 2-h, and Na_2_CO_3_ was added finally to generate fluorescent 4-methylumbelliferone. The fluorescence (Ex_360nm_/Em_450nm_) was determined (Gary and Kindell 2005), and the SAβG activity in the blank was recognized as 100%, and each treatment was expressed as relative SAβG activity (%). For in situ visualization of SAβG activity in the treated cells, the senescence detection kit was used. The treated cells were washed with 1X PBS and then fixed at room temperature for 10–15 min, washed with 1X PBS and then stained with staining solution containing X-gal (20 mg/mL) at 37 °C overnight (Burn 2012). The blue-colored cells were photographed using an inverted microscope (200-fold magnifications, ECLIPSE TS100, Nikon Instruments Inc., Tokyo, Japan).

### Gene expressions associated with ageing and anti-ageing activities by the real-time quantitative polymerase chain reaction (qPCR)

The galactose-treated SH-SY5Y cells with or without compound **1** or compound **2** pre**-**treatments together with cells in the blank were harvested, and the RNA was extracted by using the RNA isolation kit (Roche Life Science, Mannheim, Germany), and the cDNA was synthesized by the SuperScript™ II reverse transcriptase and Platinum^®^ Taq DNA polymerase (Thermo Fisher Scientific Inc.). For quantification of specific gene expressions, the real-time PCR LightCycle^®^ 480 System (Roche Life Science, Germany) was used. The cDNA, primers of the specific genes, and LightCycle^®^ 480 SYBR Green I Master (Roche Life Science, Germany) were added in the PCR tube for 45 cycles, including 10 s of denaturizing at 95 °C, 30 s of annealing at 55 °C, and 10 s of extension at 72 °C. The primers of the specific genes were synthesized by Genomics BioSci & Tech Co., Ltd (New Taipei city, Taiwan) as follows: p16, forward, 5′-CTTCCTGGACACGCTGGTG-3′, and reverse, 5′-GCATGGTTACTGCCTCTGGTG-3′ (Toupchian et al. 2018); p21, forward, 5′-GAAGTGAGCACAGCCTAG-3′, and reverse, 5′-TGCCTTCACAAGACAGAG-3′ (Yin et al. 2017); SIRT-1, forward, 5′-TGCTGGCCTAATAGAGTGGCA-3′, and reverse, 5′-CTCAGCGCCATGGAAAATGT-3′ (Yoshihara et al. 2024); GAPDH, forward, 5′-AGCCACATCGCTCAGACAC-3′, and reverse, 5′-GCCCAATACGACCAAATCC-3′ (Lu et al. 2021). Results of p16, p21, and SIRT-1 gene expressions were calculated and normalized against the GAPDH gene expressions. The gene expressions in the blank were recognized as 100% and target genes in the treated groups were expressed relative to those in the blank.

### Effects of DC-95EE or purified compounds on AChE inhibitory activities and anti-Aβ_1-42_ peptide aggregations

The AChE inhibitory activities of DC-95EE (200, 400, 1000, 1500, and 2000 μg/mL) or purified compound **1** and compound **2** (200, 400, 800 μM) were determined by following the previous reports by using synthetic AChE substrates of acetylthiocholine iodide (Sie et al. 2023a, b). An equal volume of DMSO instead of the sample solution was used in the blank group and expressed as 100% AChE activity. The absorbance changes were recorded at 405 nm for 10 min. The AChE inhibition (%) was calculated as follows: [(A405_blank _− A405_sample_)/(A405_blank_)] × 100%. The IC_50_ of the AChE inhibitory activity was calculated from each linear equation with the use of three test concentrations (400, 1000, and 1500 μg/mL and their inhibitory activities. The anti-Aβ_1-42_ peptide aggregations of DC-95EE (2.5, 5.0, 10, 15, 25, and 50 μg/mL) were determined by monitoring the fluorescent changes in ThT-bound Aβ fibrils following the previous reports (Sie et al. 2023a, b). The HFIP was used to dissolve the commercial Aβ_1-42_ peptide to prepare 1 mM stock solution, and was adjusted by 50 mM phosphate buffer (pH7.4, containing 150 mM sodium chloride and 1 mM EDTA) to 10 μM working solution. The fluorescence (Ex_440nm_/Em_486nm_) was determined at 0 h and 24 h, and the anti-Aβ aggregation (%) was calculated as [(△E_control _− △E_sample_)/(△E_control_)] × 100%. The IC_50_ of the anti-Aβ aggregation was calculated from each linear equation using three test concentrations (10, 15, and 25 μg/mL) and their inhibition capacities.

### Effects of DC-95EE or compound 2 pre-treatments on cognitive impairments in scopolamine-induced ICR mice

The protocols of animal experiments had received the approval number of No. LAC-2022-0201 by the Institutional Animal Care and Use Committee of Taipei Medical University. After 1-week acclimation, the 6-week-old ICR mice were randomly divided into four groups including blank, control, donepezil (positive control) group, and the DC-95EE group or compound **2** (schaftoside) in the two independent procedures (N = 10 heads/group in the procedure I; N = 6 heads/group in the procedure II). The 2-day passive avoidance test in the procedure I and the 3-day Morris water maze in the procedure II were used to evaluate effects of DC-95EE or the 3-day Morris water maze in the procedure II were used to evaluate effects of compound **2** (schaftoside) on the improvement of learning and memory functions in scopolamine-induced impaired cognitive mice following the previous reports (Chen et al. 2021, 2022; Yang et al. 2024). The DC-95EE were used in pre-treatments (preventive models) in the procedure I and the procedure II, which the oral administration by the gavage feeding once a day from day 1 to day 7 at concentration of 200 mg/kg of body weight (in the 0.5% PEG 400 solution). The mice in the blank, control, and donepezil groups were orally administered by the 0.5% PEG 400 solution once a day for 7 days in parallels. At day 8 and day 9 in the procedure I, and at day 8 to day 10 in the procedure II, the treatment for each mouse was the same as the past 7 days in the blank and the control; and mice in the donepezil group were treated orally the donepezil (5 mg/kg) daily once for successive 2-days or 3-days. For inductions of cognitive dysfunction, 30 min after sample oral administration, mice were intraperitoneally injected scopolamine (1 mg/kg in the one-fold diluted PBS). Mice in the blank were intraperitoneally injected the same volume of one-fold diluted PBS, and 30-min later, the learning behaviors were evaluated by the passive avoidance in the procedure I and the Morris water maze in the procedure II. All mice were sacrificed at the end of the animal experiments, and the whole brain tissues were isolated and stored immediately at − 80 °C for further determinations of AChE activities and MDA levels. For compound **2** (schaftoside) oral administration, the 25 mg/kg and 50 mg/kg (in the 0.5% PEG 400 solution) were used in pre-treatments (preventive models) in the procedure II, and the learning behaviors were evaluated by Morris water maze.

The liquid nitrogen was added to frozen brains of mice in each group, which were ground by a mortar and pestle to fine powders and then suspended in one-fold diluted PBS on the ice bath. The supernatants were collected by the centrifugation, and proteins in brain extracts were quantified by the BCA protein assay kit (Thermo Fisher Scientific Inc., USA) using bovine serum albumin as the standard protein. The MDA levels in the brain extracts were determined by BIOXYTECH^®^ MDA-586™ assay kits (Portland, OR) as previously described (Chen et al. 2022; Liu et al. 2020). The AChE activities in the mouse brain tissue extract were assayed following the previous reports (Chen et al. 2021, 2022). The donepezil (final concentration of 100 nM) was premixed with each diluted brain extracts for 30 min to determine the non-AChE-hydrolyzed DTNB-reacted products as the sample blank in the brain extracts. The AChE activities of each brain extracts were calculated as [A405_sample_-A405_sample blank_]/μg protein, and those activities in the blank group were recognized as 100%.

### Learning and memory functions in scopolamine-induced amnesiac ICR mice

The 2-day passive avoidance test in the procedure I was performed including the acquisition trial in the first day and the retention trial in the second day (Chen et al. 2021, 2022; Yang et al. 2024). The PACS-30 passive/active avoidance box (Columbus Instruments Inc., Columbus, OH) with an illuminated box and a cover-protected dark box separated by a sliding door. At the first day of passive avoidance test (the acquisition trial), each injected mouse (the 30-min after scopolamine or PBS injections) was placed in the illuminated box. While, the mouse entered the dark box, the sliding door was immediately closed and the time staying in the illuminated box (as the step-through latency, sec) was recorded. The mouse in the dark box was received an electric foot shock (0.3 mA for 3 s) via the wired metal floor and then sent back to the original cage. At the second day of passive avoidance test (the retention trial), each injected mouse was placed in the illuminated box following the first day procedure except no the electric foot shock in the dark box. The time staying in the illuminated box (as the step-through latency, sec) of each mouse in the 2nd day was recorded again. The three-successive-day Morris water maze (100 cm in diameter) using a video and computerized tracking system in the procedure II was performed following the previous reports (Han et al. 2014a, b; Chen et al. 2021). Each injected mouse was trained once a day for 125 s in the water maze to find and climb onto the submerged platform and sent back to the original cage, and the protocol was repeated again at the 2nd day. At the 3rd day, the time to find and climb onto the platform (the latency, sec) was recorded.

### Statistical analyses

The data were expressed as mean ± SD of three independent experiments, and the learning behaviors of animal experiments in the passive avoidance test and Morris water maze were expressed as mean ± SE. The statistical analysis was performed using the GraphPad Prism Software 6.0. The student *t*-test was used to analyze the differences of neuroprotective and anti-ageing activities in galactose-treated SH-SY5Y neuronal cell models, gene expressions, and step-through latency in the passive avoidance test between the control and each sample treatment or between the control and the blank, which was considered statistically significant when *P* < 0.05 *, or *P* < 0.01**, or *P* < 0.001***. The one-way analysis of variance (one-way ANOVA) followed by the post hoc Tukey’s test was used to analyze the differences among multiple groups, which the different letters were considered significantly different (*P* < 0.05) of latency in the Morris water maze under the same training day, and AChE activities and MDA levels in the brain tissues extracts.

## Results

### Neuroprotective activities of DC-95EE pre-treatments against galactose-induced senescence in SH-SY5Y neuronal cells models

**Fig. 1:**
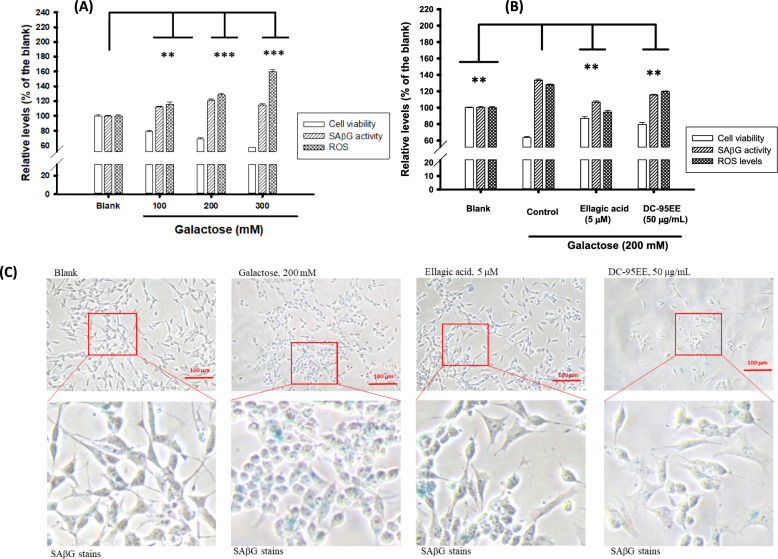
**A** The different concentrations of galactose (100, 200, and 300 mM) treatments on the cell viability, SAβG activity, and ROS levels in SH-SY5Y neuronal cell models; **B** the neuroprotective activities of DC-95EE (50 μg/mL) pre-treatments on the cell viability, SAβG activity, and ROS levels in galactose (200 mM)-treated SH-SY5Y neuronal cell models, and ellagic acid (5 μM) was used as the positive; **C** the photograms of in situ SAβG activity stains of ellagic acid (5 μM) and DC-95EE (50 μg/mL) pre-treatments in galactose-treated SH-SY5Y cells. The data were expressed as mean ± SD of three independent experiments. The student *t*-test was used to analyze the differences between the control and each sample treatment or between the control and the blank, which was considered statistically significant when *P* < 0.01** or *P* < 0.001***

Figure 1a shows the different concentrations of galactose (100, 200, and 300 mM) treatments on the SAβG activities, intracellular ROS levels, and SH-SY5Y cell viabilities. It was found that galactose treatments showed dose-dependent increases in SAβG activities and intracellular ROS levels, and resulted in the decreases in cell viabilities, which showed significantly differences compared to those in the un-treated blank (*P* < 0.01** or 0.001***). Therefore, the 200 mM of galactose was selected for further experiments. The ellagic acid was reported to exhibit anti-ageing activities in galactose-treated SH-SY5Y cells (Rahimi et al. 2018), and were used as the positive control in the present study. Figure 1b shows DC-95EE (50 μg/mL) pre-treatments against galactose (200 mM)-induced senescence in SH-SY5Y cells. It was clear that galactose treatments (200 mM, the control) for 48-h showed to reduce cell viabilities of treated SH-SY5Y cells from 100% (the blank) to about 60%, and enhance the intracellular ROS levels and SAβG activities of treated SH-SY5Y cells from 100% to about 130%, which had significant differences compared to those in the blank (*P* < 0.01**). The pre-treatment of ellagic acid (a positive control) showed to elevate cell viabilities (63.5–86.9%), reduce the intracellular ROS levels (128–95%) and SAβG activities (133–106.7%) of galactose-treated cells, and showed significant differences compared to those in the control (*P* < 0.01**). The DC-95EE pre-treatment (50 μg/mL) showed a similar pattern to those of ellagic acid in galactose-treated SH-SY5Y cells, which an increase in cell viability (63.5–79.5%) and reductions in ROS levels (128.1–119.5%) and SAβG activities (133–115%), which meant the neuroprotective activities. Figure 1c shows the photograms of SAβG activity stains of ellagic acid and DC-95EE pre-treatments in galactose-treated SH-SY5Y cells. It was clear that the galactose treatments could increase amounts of the blue stains of the in situ SAβG activities, which the treated cells were under the senescence status. The ellagic acid and DC-95EE pre-treatments showed to reduce amounts of blue stains (the locations of SAβG activities) in the galactose-induced senescent cells. It was noted that neurite lengths and pyramidal shape of SH-SY5Y cells were recovered after ellagic acid or DC-95EE pre-treatments in galactose-treated cells.

### Effects of DC-95EE on AChE inhibitory activities and anti-Aβ_1-42_ peptide aggregations and learning behaviors in scopolamine-induced mice models

**Fig. 2:**
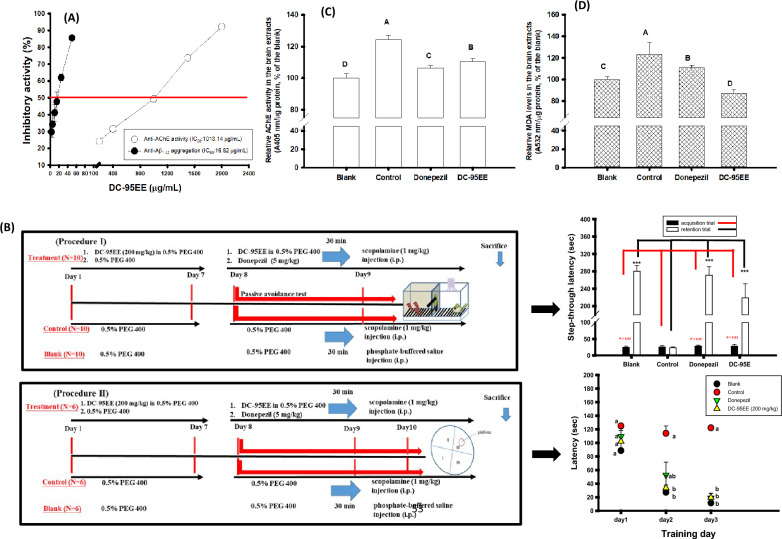
**A** Effects of different concentrations of DC-95EE on anti-AChE activities and anti-Aβ_1-42_ aggregations; **B** the animal experimental protocols in the Procedure I and the Procedure II. The preventive protocol of DC95EE (200 mg/kg) pre-treatment once a day for 7 days. For Procedure I, at day 8 and day 9, the step-through latency (sec) of acquisition trial and retention trial in passive avoidance test were used; for Procedure II, from day 8 to day 10, the latency (sec) of Morris water maze were used to evaluate the learning behaviors in the scopolamine-induced ICR mice. The AChE activity (**C**) and the MDA levels (**D**) were assayed in the brain extracts of the blank and the treated groups. Mice in the control group were injected with scopolamine only, and the donepezil (5 mg/kg) was used as the positive control. The data were expressed as mean ± SD of three independent experiments, and the learning behaviors of animal experiments in the passive avoidance test and Morris water maze were expressed as mean ± SE. The student *t*-test was used to analyze the differences of step-through latency in the passive avoidance test between the control and each sample treatment or between the control and the blank, which was considered statistically significant when *P* < 0.001***. The one-way ANOVA followed by the post hoc Tukey’s test was used to analyze the differences among multiple groups, which the different letters were considered significantly different (*P* < 0.05) of latency in the Morris water maze under the same training day, and AChE activities and MDA levels in the brain tissues extracts

Figure 2a shows effects of DC-95EE on the inhibitions of AChE activities and Aβ_1-42_ peptide aggregations in vitro, which were important factors associated with AD progressions. The present results showed that DC-95EE exhibited dose-dependent inhibitory activities against AChE activities and Aβ_1-42_ peptide aggregations with IC_50_ of 1.01 mg/mL and 16.62 μg/mL, respectively. Figure 2b shows pre-treated protocol of DC-95EE (200 mg/kg of body weight/day) for 7 days in scopolamine-induced animal models, which the learning and memory behaviors were evaluated either by the 2-day passive avoidance test (the procedure I) or by the 3-day Morris water maze (the procedure II). In the first day of acquisition trial of the procedure I, there was no significant difference of mice in the treated groups or the blank compared to the control in the step-through latency (sec) (*P* > 0.05). After being received an electric foot shock in the dark box of the first day, mice in the blank or treated with DC-95EE or donepezil (the positive control) showed a longer step-through latency (sec) in the second day of the retention trial and had significant differences compared to those in the control (*P* < 0.001***). The longer step-through latency (sec) of mice in the illuminated box at the second day compared to those in the control by pre-treating DC-95EE (200 mg/kg) daily for 7 days revealed the improved learning and memory abilities of scopolamine-treated mice.

In the first day of Morris water maze evaluation in the procedure II, mice among groups showed no significant difference (*P* > 0.05) in the required time to find the submerged platform (latency, sec). In the second day, the latency (sec) of treated mouse was generally reduced and mice in the blank and the DC-95EE group showed significant differences (*P* < 0.05) compared to those in the control. In the third day, mice in the blank, donepezil-treated and DC-95EE-treated groups showed the similar latency (sec) and lowed than those in the control, and had significant differences compared to those in the control (*P* < 0.05). The reduced latency (sec) of mice to find the submerged platform in the water maze by pre-treating DC-95EE (200 mg/kg) daily for 7 days revealed the improved abilities in spatial memory of scopolamine-treated mice.

At the end of animal experiments, mice brain extracts of different groups were used to determine AChE activities (Fig. 2c) and MDA levels (Fig. 2d) under the same extractable proteins, and the blank was recognized as 100%. The scopolamine-treated mice in the control had about 120% of the AChE activities and MDA levels and showed significant differences compared to those in the blank (*P* < 0.05). However, the donepezil-treated or DC-95EE-pre-treated mice showed to lower AChE activities and MDA levels and showed significant differences compared to those of scopolamine-treated mice in the control (*P* < 0.05).

### Neuroprotective activities of partitioned fractions of DC-95EE against galactose-induced senescence in SH-SY5Y neuronal cells models

**Fig. 3:**
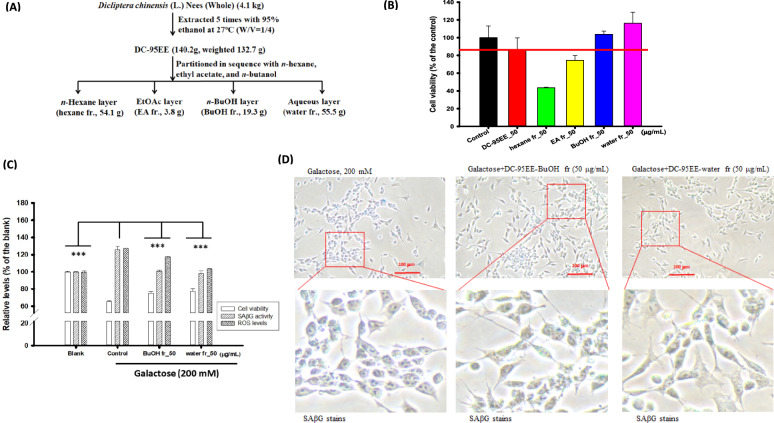
**A** The flowchart of the DC-95EE extracted from DC, which were partitioned sequentially to get *n*-hexane fraction, EA fraction, BuOH fraction, and aqueous layer. **B** Effects of DC-95EE and its partitioned fractions (*n*-hexane fraction, EA fraction, BuOH fraction, and water fraction, 50 μg/mL) on cell viabilities of SH-SY5Y neuronal cell models detected by MTT methods. The 0.1% DMSO was used instead of sample solution in the control. **C** The neuroprotective activities of BuOH fractions and water fraction (50 μg/mL) pre-treatments on the cell viability, SAβG activity, and ROS levels in galactose (200 mM)-treated SH-SY5Y neuronal cell models; **D** The photograms of in situ SAβG activity stains of BuOH fractions and water fraction (50 μg/mL) pre-treatments in galactose-treated SH-SY5Y cells. The data were expressed as mean ± SD of three independent experiments. The student *t*-test was used to analyze the differences between the control and each sample treatment or between the control and the blank, which was considered statistically significant when *P* < 0.001***

Figure 3a shows the flowchart of the DC-95EE extracted from DC, which were partitioned sequentially to get *n*-hexane fraction, EA fraction, BuOH fraction, and aqueous layer. The DC-95EE recovery was about 3.42% from DC. The recovery of partitioned fractions of *n*-hexane fraction, EA fraction, BuOH fraction, and aqueous layer of DC-95EE, respectively, were 40.77%, 2.86%, 14.54%, and 41.82%. Figure 3b shows the effects of DC-95EE and its partitioned fractions on cell viabilities of SH-SY5Y neuronal cells models, and the control (DMSO-treated cells) was recognized as 100%. The cell viabilities of DC-95EE, *n*-hexane fraction, EA fraction, BuOH fraction, and aqueous layer, respectively, were (85.91 ± 13.85) %, (43.52 ± 0.62) %, (74.47 ± 5.29) %, (103.85 ± 3.56) %, and (116.23 ± 12.49) % under the same concentration of 50 μg/mL. The *n*-hexane fraction and EA fraction of DC-95EE showed higher cyto-toxicities than other two fractions in SH-SY5Y neuronal cell models, therefore, BuOH fraction and aqueous layer were used for neuroprotection activity assays. Figure 3c shows the pre-treatments of BuOH fraction and aqueous layer (50 μg/mL) of DC-95EE in the cell viability, SAβG activities, and ROS levels against galactose (200 mM)-induced senescence in SH-SY5Y cells. It is clear that the BuOH fraction and aqueous layer (50 μg/mL) of DC-95EE can elevate cell viability, reduce the SAβG activities, and lower ROS levels of galactose-treated cells. The pre-treatment of BuOH fraction showed to elevate cell viabilities (65.54–75.26%), reduce the SAβG activities (125.68–100.66%), and lower intracellular ROS levels (127.12–117.36%) of galactose-treated cells, and showed significant differences compared to those in the control (*P* < 0.001***). The pre-treatment of aqueous layer showed to elevate cell viabilities (65.54–77.38%), reduce the SAβG activities (125.68–97.93%), and lower intracellular ROS levels (127.12% to103.42%) of galactose-treated cells, and showed significant differences compared to those in the control (*P* < 0.001***). These results meant the neuroprotective activities of BuOH fraction and aqueous layer in vitro. Figure 3d shows the photograms of in situ SAβG activity stains of the pre-treatments of BuOH fraction and aqueous layer in galactose-treated SH-SY5Y neuronal cell models. The blue SAβG activity stains revealed the senescent status in galactose-treated SH-SY5Y cells. The BuOH fraction and aqueous layer of DC-95EE pre-treatments showed to reduce amounts of blue stains (the locations of SAβG activities) in the galactose-induced senescent cells. It was also noted that neurite lengths of galactose-treated SH-SY5Y cells were recovered after pre-treatments of these two fractions.

### Isolations and identifications of natural compounds from BuOH fraction of DC-95EE and HPLC fingerprints of DC-95EE

**Fig. 4:**
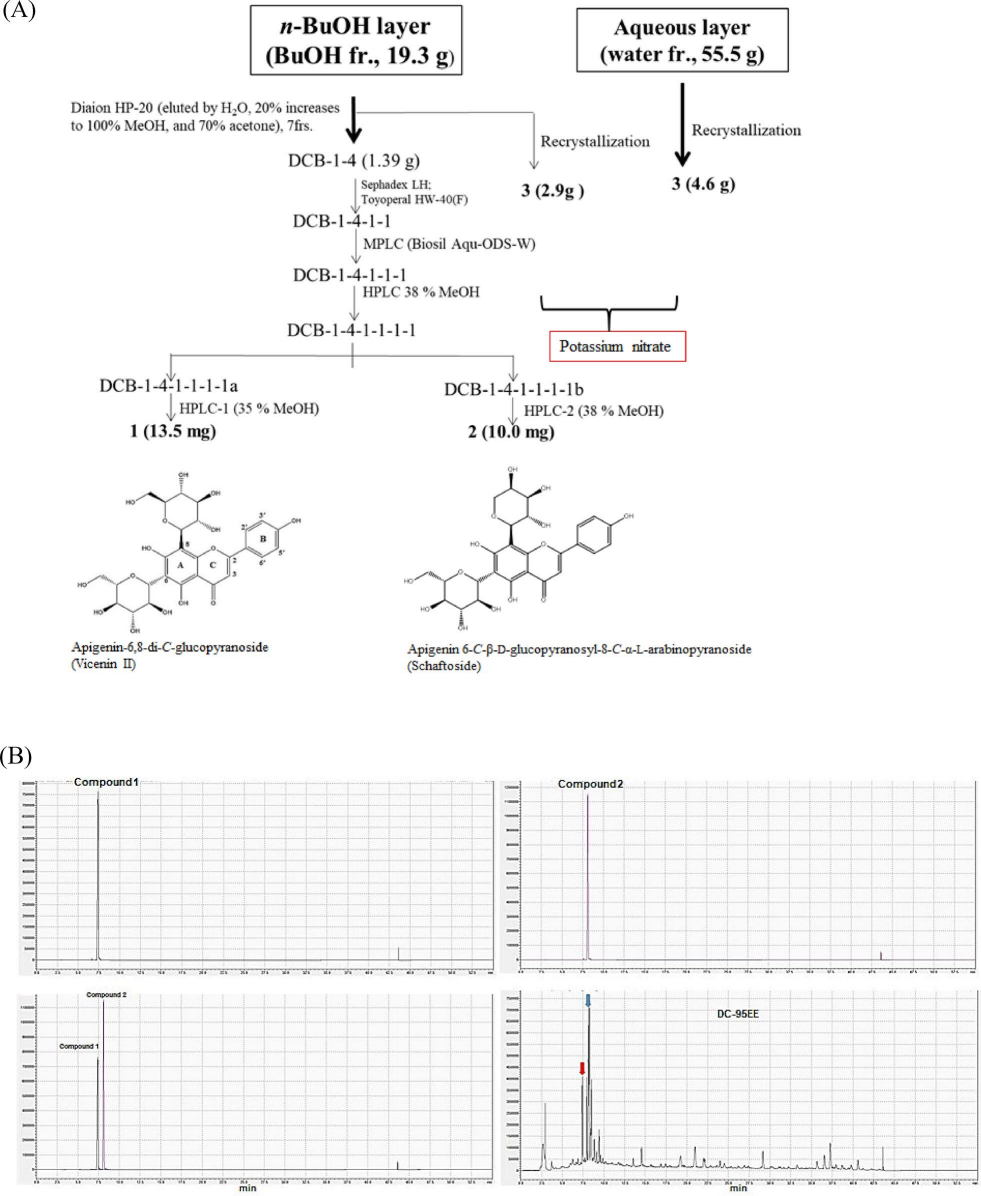
**A** The flowchart of isolation protocol of compound **1** and compound **2** and the structures from the BuOH fractions of DC-95EE. The compound **3** was repeatedly re-crystallized from precipitates of BuOH fraction and aqueous layer of DC-95EE. **B** The HPLC fingerprints of DC-95EE. The HPLC chromatograms of compound **1**, compound **2**, and the overlapping figures of compound **1** and compound **2**. The arrows indicated the positions of compound **1** and compound **2** in the DC-95EE. The analytical Galaksil EF-C18 (5 μm, 250 × 4.6 mm) was performed using Shimadzu SCL-40 chromatography system with a gradient elution program was set in solvent mixtures of distilled water and acetonitrile as follows: water/acetonitrile, 90/10, at 0–35 min; 0/100, at 35–40 min; 90/10, at 40.01–55 min. The concentrations of DC-95EE, compound **1**, and compound **2*,*** respectively, were 100 mg/mL, 1 mg/mL, and 1 mg/mL, and 10 μL was injected for analysis. The flow rate was 1.0 mL/min and the wavelength was set at 335 nm for monitoring. The identified compounds included compound **1** (vicenin II, 7.415 min) and compound **2** (schaftoside, 8.112 min)

Figure 4a shows the flowchart of natural compound isolations from the BuOH fraction of DC-95EE. Two purified compounds (compound **1** and compound **2**) were isolated and identified from the BuOH fraction of DC-95EE, and one compound (compound **3**) from repeated crystallizations from the white needle-like precipitations of BuOH fraction and aqueous layer of DC-95EE. All the spectral data in the ^1^H-NMR, ^13^C-NMR, HSQC-NMR, and HMBC-NMR of compounds **1** (Supplementary Figs. S1A, S1B, S1C, S1D) and compound** 2** (Supplementary Figs. S2A, S2B, S2C, S2D) were summarized in Table 1. The *m/z* of quasi-molecular ion [M + H]^+^ of compound **1** (Supplementary Fig. S1E) and compound **2** (Supplementary Fig. S2E) were 595.17 and 565.15 with the deduced molecular formula of C_27_H_34_O_15_ and C_26_H_28_O_14_, respectively, by the TOF–MS, which were compatible with those of apigenin-6,8-di-*C*-glucopyranoside (vicenin II, compound **1**) (Yoshizaki et al. 1987; Velozo et al. 2009), and apigenin 6-*C*-β-d-glucopyranosyl-8-*C*-α-l-arabinopyranoside (schaftoside, compound **2**) (Xie et al. 2003). The compound **3** was repeatedly re-crystallized from precipitates of BuOH fraction and aqueous layer of DC-95EE. The supplementary Fig. S3 shows X-ray diffraction data of **compound 3**, (A) the structure of crystal system, (B) average bond length, and (C) summary of crystal data. Compound **3** was proposed to be potassium nitrate (KNO_3_ with formula weight of 101.11 Da) (Freney et al. 2009), which might be as the fertilizers for its high amounts existed in the plants. Figure 4b shows the HPLC fingerprints of DC-95EE. Comparing with the standard compounds, arrows indicate the positions of compound **1** (7.415 min) and compound **2** (8.112 min) in the DC-95EE. The supplementary Fig. S4 shows the HPLC chromatogram of the overlapping compound **1** (vicenin II) and compound **2** (schaftoside) with the DC95-EE by different elution programs. It was found that the overlapping time of compound **1** and compound **2** with DC-95EE, respectively, was 11.426 min and 21.341 min. It was clear compound **1** and compound **2** can be detected in the DC-95EE. For calculations of compound **1** and compound **2** in the DC-95EE, the two-fold dilution from 500 to 1.953 μg/mL were prepared to plot the standard curves of concentrations versus area in HPLC chromatograms. It was found that compound **1** (vicenin II) and compound **2** (schaftoside), respectively, accounted for about 2.53% and 8.17% in the DC-95EE by HPLC.

**Table 1:** The ^1^H- and ^13^C-NMR spectral data of two purified compounds from the BuOH fractions of the DC-95EE (δ values, in methanol-*d*4, *J* in Hz, 500 MHz ^1^H and 125 MHz ^13^C)

Compound** 1:** apigenin-6,8-di-*C*-glucopyranoside (vicenin II)				Compound** 2:** apigenin-6-*C*-β-d-glucopyranosyl-8-*C*-α-l-arabinopyranoside (schaftoside)			
	
Position	δ_C_	δ_H_ (mult, *J* in Hz)	HMBC	Position	δ_C_	δ_H_ (mult, *J* in Hz)	HMBC
2	163.7			2	163.8		
3	102.5	6.78 (1H, *s*)	C-1′, C-2, C-4, C-10	3	102.3	6.79 (1H, *s*)	C-1′, C-2, C-4, C-10
4	182.0			4	182.2		
5	158.8			5	159.5		
6	107.6			6	108.5		
7	161.2			7	162.2		
8	105.3			8	104.5		
9	155.1			9	154.3		
10	103.2			10	103.1		
1′	121.6			1′	121.2		
2′, 6′	128.9	8.00 (2H, *br s*)	C-2, C-4′	2′, 6′	129.1	8.10 (2H, *br s*)	C-2, C-4′, C-3′, C-5′
3′, 5′	115.9	6.93 (2H, *d*, *J* = 6.7 Hz)	C-1′, C-4′	3′, 5′	116.0	6.90 (2H, *d*, *J* = 8.8 Hz)	C-1′, C-4′
4′	161.2			4′	161.1		
6-Glu-1″	74.1	4.77 (1H, *m*)		6-Glu-1″	75.5	4.69 (1H, *d*, *J* = 9.0 Hz)	C-6, C-2”
2″	71.8	3.55 (1H, *m*)		2″	70.7	3.87 (1H,* d*, *J* = 10.4 Hz)	
3″	78.0	3.27 (1H, *m*)		3″	78.6	3.23 (1H, *m*)	C-4”
4″	69.2	3.34 (1H, *m*)		4″	70.0	3.22 (1H, *m*)	
5″	81.9	3.22 (1H, *m*)		5″	81.4	3.22 (1H, *m*)	
6″	59.9	3.64 (2H, *m*)		6″	60.9	3.66 (1H,* d*, *J* = 11.4 Hz)	C-4″, C-5”
						3.48 (1H, *m*)	
8-Glu-1‴	73.4	4.77 (1H, *m*)		8-Ara-1‴	74.9	4.76 (1H, *m*)	C-8
2‴	71.0	3.87 (1H, *m*)		2‴	68.8	4.06 (1H, *m*)	
3‴	78.9	3.27 (1H, *m*)		3‴	74.6	3.48 (1H, *m*)	C-4″’
4‴	70.6	3.37 (1H, *m*)		4‴	68.8	3.84 (1H,* d*, *J* = 10.5 Hz)	C-3″’, C-5″’
5‴	80.9	3.28 (1H, *m*)		5‴	70.7	3.87 (1H,* d*, *J* = 10.5 Hz)	
						3.66 (1H,* d*, *J* = 11.4 Hz)	
6‴	61.3	3.51 (1H, *m*)					
		3.74 (1H, *m*)					
5-OH		13.71 (1H, *br s*)		5-OH		13.81 (1H, *br s*)	

### Neuroprotective activities of compound 1 and compound 2 against galactose-induced senescence in SH-SY5Y neuronal cells models

**Fig. 5:**
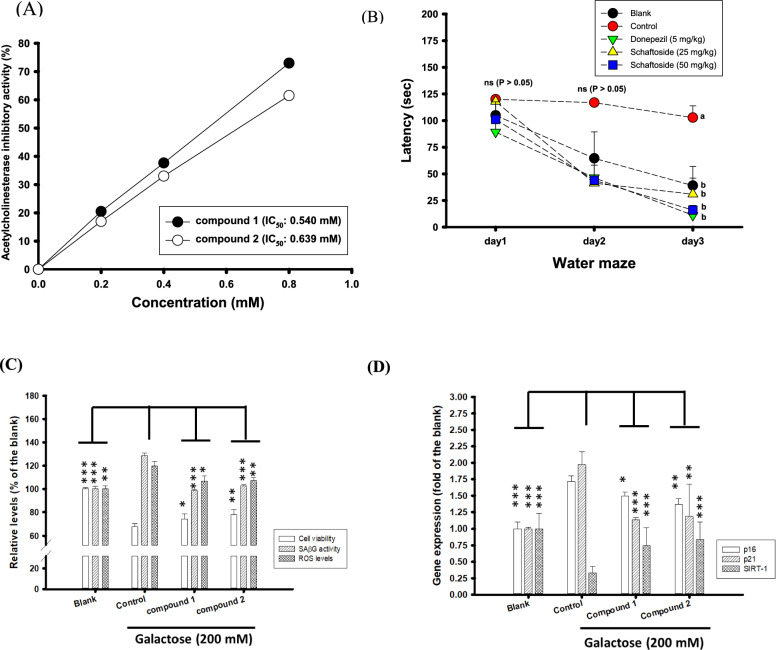
**A** Effects of different concentrations of compound **1** and compound **2** on anti-AChE activities; **B** The preventive protocol of compound 2 (25 and 50 mg/kg) pre-treatment once a day for 7 days and evaluated by procedure II. For Procedure II, from day 8 to day 10, the latency (sec) of Morris water maze were used to evaluate the learning behaviors in the scopolamine-induced ICR mice. **C** The neuroprotective activities of compound **1** and compound **2** (100 μM) pre-treatments on the cell viability, SAβG activity, and ROS levels in galactose (200 mM)-treated SH-SY5Y neuronal cell models; **D** Effects of compound **1** and compound **2** (100 μM) pre-treatments on the gene expressions associated with ageing (p16 and p21) and anti-ageing (SIRT-1) activities by the qPCR in galactose (200 mM)-treated SH-SY5Y neuronal cell models. Mice in the control group were injected with scopolamine only, and the donepezil (5 mg/kg) was used as the positive control. The learning behaviors of animal experiments in Morris water maze were expressed as mean ± SE. The one-way ANOVA followed by the post hoc Tukey’s test was used to analyze the differences among multiple groups, which the different letters were considered significantly different (*P* < 0.05) of latency in the Morris water maze under the same training day

Figure 5a shows effects of compound **1** (vicenin II) and compound **2** (schaftoside) on the AChE inhibitory activities in vitro. The present results showed that compound **1** (vicenin II) and compound **2** (schaftoside) exhibited dose-dependent AChE inhibitory activities with IC_50_ of 0.540 mM and 0.639 mM, respectively. As compound **2** (schaftoside) accounted for about 8.17% of DC-95EE and had higher amounts than compound 1 (vicenin II), therefore, compound **2** (schaftoside) was selected to evaluate learning behaviors in scopolamine-induced mice models. Figure 5b shows learning behaviors of compound **2** (schaftoside, 25 and 50 mg/kg of body weight/day) for 7-day pre-treatments in scopolamine-induced learning dysfunction mice models, which the learning and memory behaviors were evaluated by the 3-day Morris water maze (the procedure II, Fig. 2b). In the first and second days of Morris water maze evaluations, mice among groups showed no significant difference (*P* > 0.05) in the required time to find the submerged platform (latency, sec). In the third day, mice in the blank, donepezil-treated and compound **2**-treated groups showed the lowed latency (sec) than those in control, and had significant differences compared to those in the control (*P* < 0.05). The reduced latency (sec) of mice to find the submerged platform in the water maze by pre-treatments of compound **2** at either 25 or 50 mg/kg/day for 7 days revealed the improved abilities in spatial memory of scopolamine-treated mice. Figure 5c shows the pre-treatments of purified compound **1** (vicenin II) and compound **2** (schaftoside) in the cell viability, SAβG activities, and ROS levels against galactose (200 mM)-induced senescence in SH-SY5Y neuronal cell models. The cells without galactose treatments (the blank) were recognized as 100%. These two isolated pure compounds at concentrations of 100 μM can elevate cell viability, reduce the SAβG activities, and lower ROS levels of galactose-treated SH-SY5Y cells. The pre-treatment of compound **1** (vicenin II) showed to elevate cell viabilities (67.87–74.24%), reduce the SAβG activities (128.74–98.51%) and intracellular ROS levels (119.53–106.89%) of galactose-treated cells, and showed significant differences compared to those in the control (*P* < 0.05*, 0.001***). The pre-treatment of compound **2** (schaftoside) showed to elevate cell viabilities (67.87–77.94%), reduce the SAβG activities (128.74–102.59%), and lower intracellular ROS levels (119.53–107.222%) of galactose-treated cells, and showed significant differences compared to those in the control (*P* < 0.01**, 0.001***). These results meant the neuroprotective activities of these two isolated compounds against oxidative stress in cell models. Figure 5d shows the gene expressions by qPCR in senescence-associated (p16, p21) and anti-senescence-associated (SIRT-1) with or without pre-treatments of compound **1** (vicenin II) and compound **2** (schaftoside) in galactose-treated SH-SY5Y cells. The targeted gene expressions in SH-SY5Y cells without galactose treatments (the blank) were recognized as the basal level (one-fold). The galactose treatments (200 mM, the control) showed to elevate senescence-associated gene expressions (p16, 1–1.72-folds; p21, 1–1.97-folds) and lower anti-senescence-associated gene expressions (SIRT-1, 1–0.33-folds), and showed significantly different compared to those in the blank (*P* < 0.001***). The pre-treatments of compound **1** (vicenin II) in galactose-treated SH-SY5Y showed to reduce senescence-associated gene expressions (p16, 1.72- to 1.49-folds; p21, 1.97- to 1.14-folds) and elevate anti-senescence-associated gene expressions (SIRT-1, 0.33- to 0.74-folds) and showed significantly different compared to those in the control (*P* < 0.05*, 0.001***). The pre-treatments of compound **2** (schaftoside) in galactose-treated SH-SY5Y showed to reduce senescence-associated gene expressions (p16, 1.72- to 1.37-folds; p21, 1.97- to 1.18-folds) and elevate anti-senescence-associated gene expressions (SIRT-1, 0.33- to 0.84-folds) and showed significantly different compared to those in the control (*P* < 0.01**, 0.001***).

## Discussion

The present results revealed for the first time that the DC-95EE, and the isolated compound **1** (vicenin II) and compound **2** (schaftoside) from BuOH fractions exhibited anti-ageing activities in galactose-induced oxidative stress SH-SY5Y neuronal cell models and the oral administration of DC-95EE and compound **2** (schaftoside) daily for 7-days also exhibited the improved learning and memory activities in scopolamine-induced impaired cognitive mice. The compound **1** (vicenin II) and compound **2** (schaftoside), respectively, accounted for about 2.53% and 8.17% in the DC-95EE by HPLC quantifications. It was possible that compound **1** (vicenin II) and compound **2** (schaftoside) acted as active compounds in the DC-95EE for anti-ageing activities and will need further investigations. Several compounds have been isolated from DC plants grown in China (Luo et al. 2002; Gao et al. 2006, 2007a, b; Huang et al. 2012; Zou et al. 2012), however, the vast amounts of compound **1** (vicenin II) and compound **2** (schaftoside) were identified and isolated from the whole DC plants grown in Taiwan, which the possibilities of geographical environments resulted in differences of chemical compositions will need investigations further, such as by LC–MS/MS.

Xu et al. (2017) firstly reported that the polysaccharides extracted from DC plants exhibited free radical scavenging activities in vitro, and the oral administration of DC polysaccharide had anti-ageing activities in galactose-induced aged mice, which the total antioxidant capacities and antioxidant enzyme activities were apparently increasing in vivo. The present study showed DC-95EE not only had neuroprotective activities to reduce intracellular ROS levels and SAβG activities to elevate cell viabilities in galactose-treated SH-SY5Y neuronal cell models (Fig. 1), but also exhibited the activities of anti-AChE and anti-Aβ peptide aggregations in vitro, and the improved learning and memory activities in scopolamine-induced impaired cognitive mice (Fig. 2). Owing higher cyto-toxicities of hexane and EA fractions of DC-95EE toward SH-SY5Y neuronal cell models than those of BuOH fractions, therefore, BuOH fractions were selected for isolating active compounds for anti-ageing activities. Two active *C*-glycosyl flavones were isolated from BuOH fractions and identified as vicenin II (apigenin-6,8-di-*C*-glucopyranoside, compound **1**) and schaftoside (apigenin 6-*C*-β-d-glucopyranosyl-8-*C*-α-l-arabinopyranoside, compound **2**). These two pure compounds showed not only neuroprotective activities to reduce intracellular ROS levels and SAβG activities to elevate cell viabilities in galactose-treated SH-SY5Y neuronal cell models (Fig. 5c). The high amounts of compound **2** (schaftoside) in the DC-95EE showed dose-dependent anti-AChE activities in vitro, and also exhibited the improved learning and memory activities in scopolamine-induced impaired cognitive mice by oral administrations (Fig. 5), which may be the active compound responsible for anti-ageing and attenuating cognitive declines of DC-95EE.

In literatures, vicenin II and schaftoside have been reported several biological activities in vitro and in vivo. The treatments of vicenin II (0.3, 0.6, 1.2 mg/kg, 12 h after lipopolysaccharide injection) had been reported to exhibit hepatoprotective activities and elevated survival rates against lipopolysaccharide-induced liver failure and lethality in mice (Lee and Bae 2020). The daily treatments of vicenin II (10 mg/kg) for 12 weeks could mitigate osteoporosis by restoring tabular thickness and area in ovariectomized rats (Zhang et al. 2020). The oral administration of vicenin II (50 mg/kg) daily for 7 days showed effectively suppresses dextran sulfate sodium-induced ulcerative colitis in C57BL/6 mice by attenuating expressions of iNOS and COX-2 and reduced bleeding scores (Yin et al. 2019). Using human dermal fibroblasts as cell models, vicenin II pre-treatments significantly reduced the UVB-induced cytotoxicity, intracellular ROS generation, and apoptotic morphological changes (Duan et al. 2019). The schaftoside isolated from 75% ethanol extracts of *Clinacanthus nutans* showed to hepatoprotective activities against CCl_4_-induced liver injuries in mice via amplifications of Nrf2 expression and triggered the Nrf2/GPX4 pathway to reduce oxidative stress levels and attenuate liver fibrosis (Yu et al. 2024). Using zebrafishes as animal models, the schaftoside pre-treatments showed to suppress seizure-like behaviors and prolonged onsets of seizures in pentylenetetrazol-induced epileptic models (Dang et al. 2021).

It has been reported that the reaction rate of D-galactose was about 4.7-fold of that of D-glucose in the use of hemoglobin as targeted proteins for AGE formations in vitro (Burn and Higgins 1981), and the intracellular ROS was produced by interactions of AGEs with AGE receptors via activations of NADPH oxidase (Calcutt et al. 2009), which the excess ROS productions higher than those be scavenged in cells could link the theory of “free radical theory of aging” (Cui et al. 2012). Therefore, some natural compounds were administered orally to galactose-induced oxidative stress animal models to investigate the changes of oxidative status and in advance to perform the improvements of the learning and memory functions by passive avoidance test and Morris water maze. While, the daily galactose injections to rodents for 6–10 weeks to induce oxidative stress was a frequent ageing model, which the ROS and AGEs generations might destroy spatial memories (Kumar et al. 2011). The oral administration of yam dioscorin (80 mg/kg) to D-galactose-induced oxidative damages of BALB/c mice daily for 6 weeks had shown not only to elevate antioxidant status by lowering MDA levels and elevating oxygen radical absorbance capacity (ORAC) activities of plasma, liver and brain extracts, but also to improve learning and memory behaviors by shortening the time to find the submerged platform (the latency, sec) in Morris water maze (Han et al. 2014a). Two minor curcuminoids of the demethylcurcumin or tetrahydroxycurcumin interventions for 4 weeks to the 8-week d-galactose-induced oxidative stress of BALB/c mice had showed to lower MDA levels of plasma and brain extracts, and iNOS protein expressions, and elevate antioxidant activities of serum ORAC (Liu et al. 2019). It was also found that the demethylcurcumin acted as a radical scavenger and/or inducer to elevate antioxidant defense systems in 6-OHDA-treated SH-SY5Y neuronal cells to reduce cell death, and exhibited anti-AChE activities and anti-Aβ aggregations in vitro and improved learning behaviors in scopolamine-induced amnesia ICR mice evaluated by passive avoidance and Morris water maze (Liu et al. 2020), which the DC-95EE and compound **2** (schafoside) exhibited the similar above-mentioned activities. The older age had been reported to be the greatest risk factor of Alzheimer’s disease (National Institute on Aging 2025). The whole plant of fresh DC are edible vegetables and the dried DC powders as tea materials in Taiwan. Therefore, DC might be beneficial in anti-ageing and anti-cognitive declines and will need further investigations.

The term “cellular senescence”, formally described at 1961 (Hayflick and Moorhead 1961), is a complex physiological condition, in which cells are under the non-proliferative but viable state to encounter environmental stressors or reflect intracellular status, including telomere attrition, treatments of cancer drugs or irradiation, nutrient deprivation or high-fat diets, oxidative stress, mitochondrial dysfunction, and inhibitors of cyclin-dependent kinases (CDKs), which the cell-cycle arrest is characterized as a general procedure. The retinoblastoma family membranes are phosphorylated by CDK2, CDK4, CDK6 to participated the cell-cycle progression; and both p21 (as the CDK2 inhibitor) and p16 (as the CDK4/6 inhibitor) up-regulated and accumulated in the senescent cells, among which p21 and p16 were involved in prolonged inhibitions of CDKs-cyclin activities to regulate cell-cycle arrests (van Deursen 2014; Gorgoulis et al. 2019). While, sirtuins (SIRT-1) are NAD^+^-dependent deacetylases that consumed NAD^+^ to promote DNA repair and were associated with longevity and metabolic regulations. The NAD^+^ levels were reported to decrease with age, and deficiencies of SIRT-1 might weaken mitophagy functions which were associated with age-related mitochondrial dysfunctional diseases including Alzheimer disease (Hou et al. 2019). Comparing with the normal ageing, the neurodegenerative disorders of AD and Parkinson’s disease showed to enhance p16 and p21 expressions, as the hallmarks of neuronal cell senescence (Tan et al. 2014). The treatments of 2,3,7,8-tetrachlorodibenzo-*p*-dioxin (TCDD), as an environmental pollutant, showed to promote intracellular ROS levels, mitochondrial dysfunctions and SAβG activity in situ, and induce p16 and p21 senescence-associated protein expressions in PC12 rat pheochromocytoma and SH-SY5Y human neuroblastoma cell models, and the antioxidant of *N*-acetylcysteine treatments showed to ameliorate TCDD-induced senescence and damages in treated neuronal cell models (Wan et al. 2014). In the present study, the treatments of galactose in the SH-SY5Y cell models showed similar results as TCDD treatments in elevating intracellular ROS levels and SAβG activity and up-regulating p16 and p21 gene expressions and down-regulating SIRT-1 gene expressions. The pretreatments of DC-95EE, and the isolated compound **1** (vicenin II) and compound **2** (schaftoside) showed to attenuate cell senescence and cell deaths via reductions of intracellular ROS levels and SAβG activities, and downregulations of p16 and p21 gene expressions and up-regulations of SIRT-1 gene expressions.

The learning behaviors of scopolamine-treated mice in the present study were evaluated by two general methods, one was the Morris water maze based on the spatial memory tasks, and the other was the passive avoidance test based on a freezing response of contextual fear conditioning by aversive stimulus of the foot shock, which the effective communications between the hippocampus and amygdala were important for the former, and the intact basolateral amygdala complex were essential for the latter (Bryan et al. 2009; Curzon et al. 2009). It has reported that scopolamine-induced cognitive mice pre-treated with resveratrol tetramer of vitisin A or co-treated with AChE inhibitor of donepezil showed to improve learning behaviors in passive avoidance tests, which both treatments not only to lower the increased AChE and MDA levels, but also to stimulate protein expressions of brain-derived neurotrophic factor (BDNF) and its ligand of TrkB for modulating synaptic plasticity (Chen et al. 2022). The improved learning behaviors of mice after DC-95EE or compound **2** (schaftoside) treatments in the present study might be also to restore BDNF/TrkB signaling pathway to recover synaptic functions and had protective roles against neuron cell apoptosis and hippocampus-mediated learning and memory functions, which will need further investigations.

## Conclusions

**Fig. 6:**
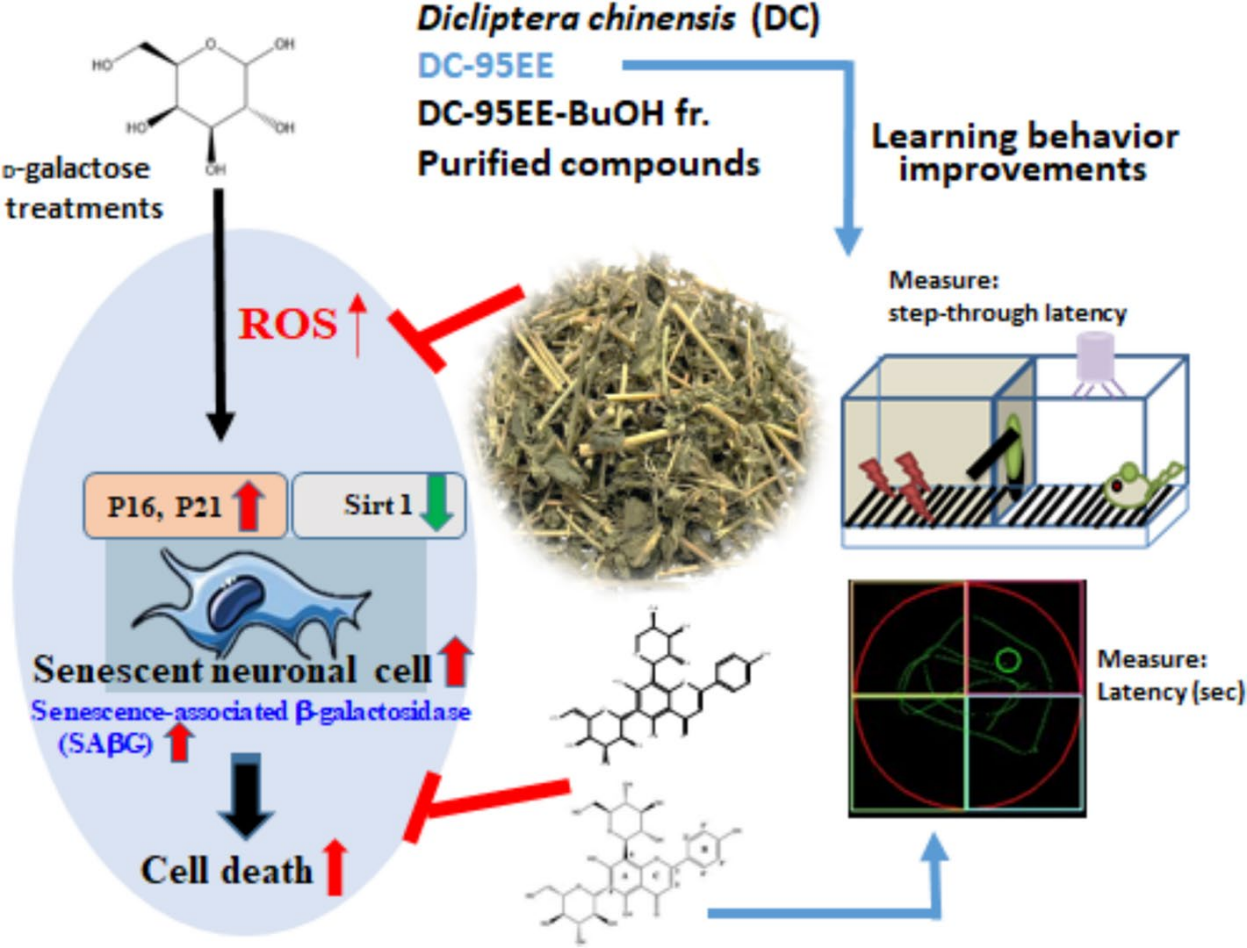
The summary of anti-ageing and attenuating cognitive declines of *Dicliptera chinensis* (L.) Nees extracts and purified compounds in vitro and scopolamine-induced cognitive dysfunction mice

In this study, the dried powders of whole DC were used as materials to investigate anti-ageing activities of DC-95EE, BuOH fractions, and the two purified compounds in SH-SY5Y neuronal cell models. It was found that DC might be beneficial in anti-ageing and anti-cognitive declines and will need further investigations. The summary of the present study is shown in the Fig. 6. The DC-95EE exhibited dose-dependent inhibitory activities against AChE and Aβ_1-42_ peptide aggregations, and the oral administrations of DC-95EE or compound **2** (schaftoside) to scopolamine-induced cognitive dysfunction mice showed the improved learning behaviors. The galactose treatments could induce cell senescence and cell deaths of neuronal cells via elevations of intracellular ROS levels and SAβG activities, and up-regulated p16 and p21 gene expressions and down-regulated SIRT-1 gene expressions. While, the pre-treatments of DC-95EE, BuOH fraction, and isolated compound **1** (vicenin II) and compound **2** (schaftoside) from BuOH fractions showed neuroprotective activities to reduce cell senescence and cell deaths of neuronal cells via reductions of intracellular ROS levels and SAβG activities, and down-regulated p16 and p21 gene expressions and up-regulated SIRT-1 gene expressions. The fresh whole DC are recognized as vegetables and also be herbal medicines. It will be beneficial to develop DC extracts as anti-ageing functional foods for daily uses.

## Supplementary Information

**Additional file 1. Fig. S1**. Structural identification of compound 1 by **A** 1H-NMR, **B** 13C-NMR, **C** HSQC-NMR, **D** HMBC-NMR, and **E** high resolution TOF-MS. **Fig. S2** Structural identification of compound 2 by **A** 1H-NMR, **B** 13C-NMR, **C** HSQC-NMR, **D** HMBC-NMR, and **E** high resolution TOF-MS. **Fig. S3** **A** The structure of crystal system, **B** average bond length, and **C** summary of crystal data of compound 3. **Fig. S4** The HPLC chromatogram of the overlapping compound 1 and compound 2 with the DC95-EE.

## Data Availability

All data has been provided with the manuscript. If any additional information is required, then the corresponding author can be contacted.
