# Supplementary material for: Anti-ageing and attenuating cognitive declines of Dicliptera chinensis extracts and purified compounds in vitro and in scopolamine-induced cognitive dysfunction mice
Source: Bot Stud. 2025 Sep 29;66:28. doi: 10.1186/s40529-025-00478-8 (PMC12480319; doi:10.1186/s40529-025-00478-8)

**Supplementary**

**Anti-ageing and attenuating cognitive declines of *Dicliptera chinensis* extracts and purified compounds i*n vitro* and in scopolamine-induced cognitive dysfunction mice models**

Yi-Yan Sie^1^, Mei-Hsien Lee^1,2,*^, Wen-Chi Hou^1,2,*^

^1^Ph.D. Program in Clinical Drug Development of Herbal Medicine, Taipei Medical University, Taipei 110, Taiwan

^2^Graduate Institute of Pharmacognosy, Taipei Medical University, Taipei 110, Taiwan

***Correspondence**

Prof. Hou, Wen-Chi

E-mail address: wchou@tmu.edu.tw

Or

Prof. Lee, Mei-Hsien

E-mail address: lmh@tmu.edu.tw

**Figure S1.** Structural identification of compound **1** by (A) ^1^H-NMR, (B) ^13^C-NMR, (C) HSQC-NMR, (D) HMBC-NMR, and (E) high resolution TOF-MS

(A)


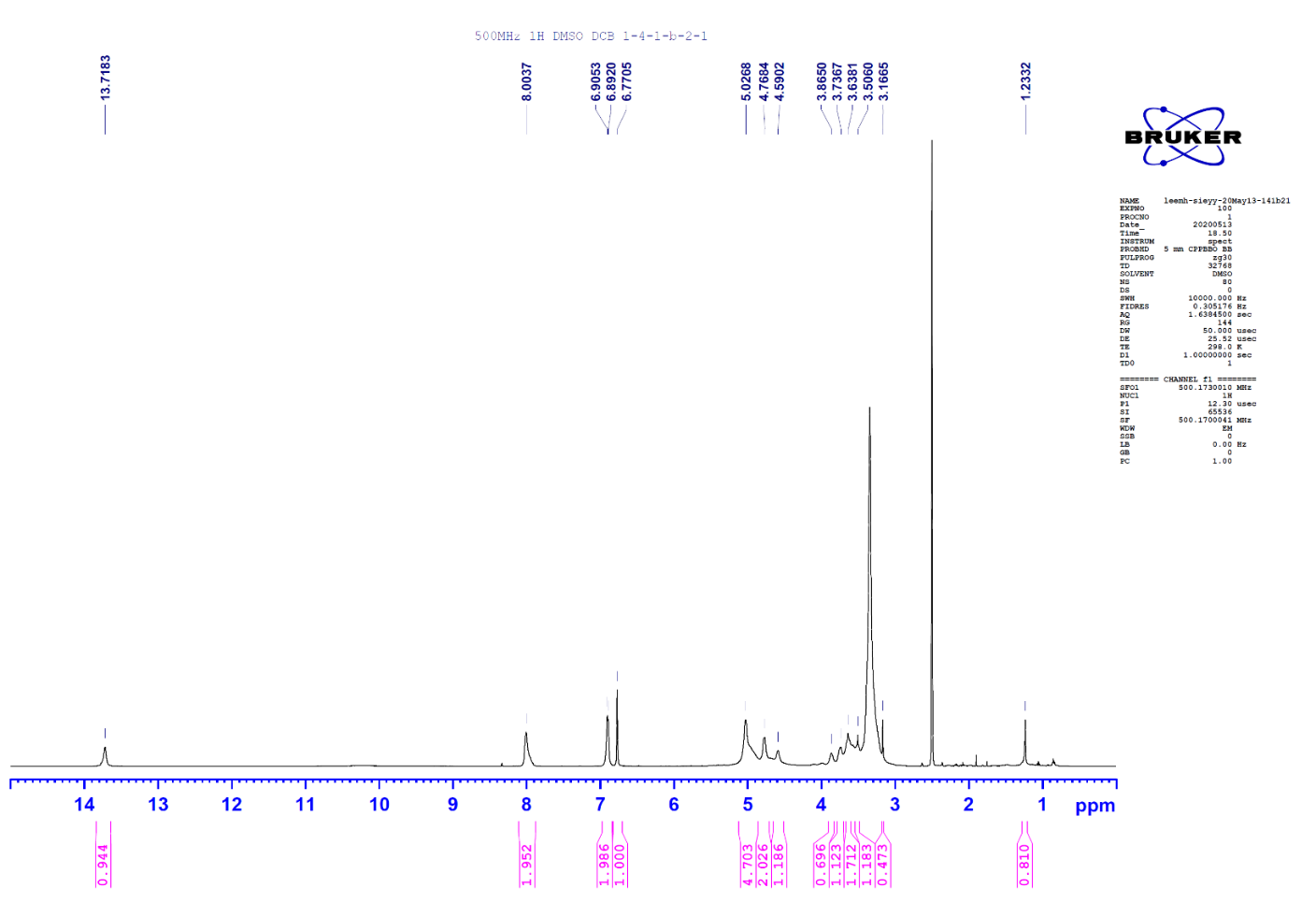


(B)


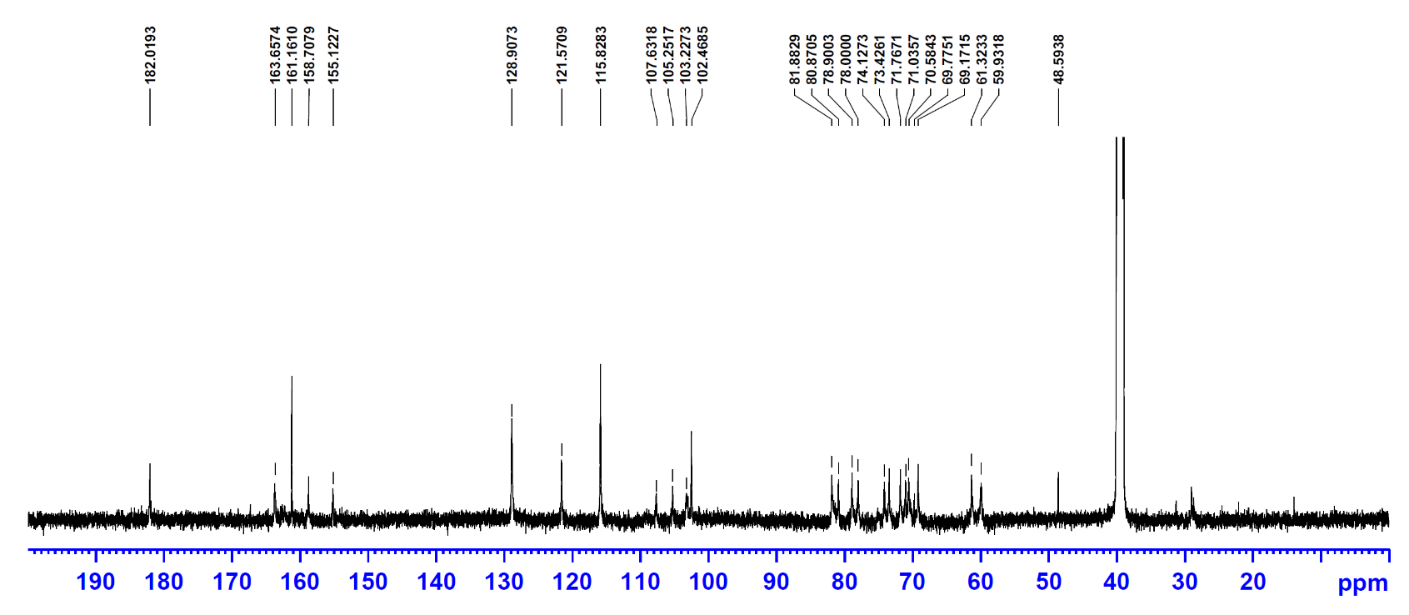


(C)


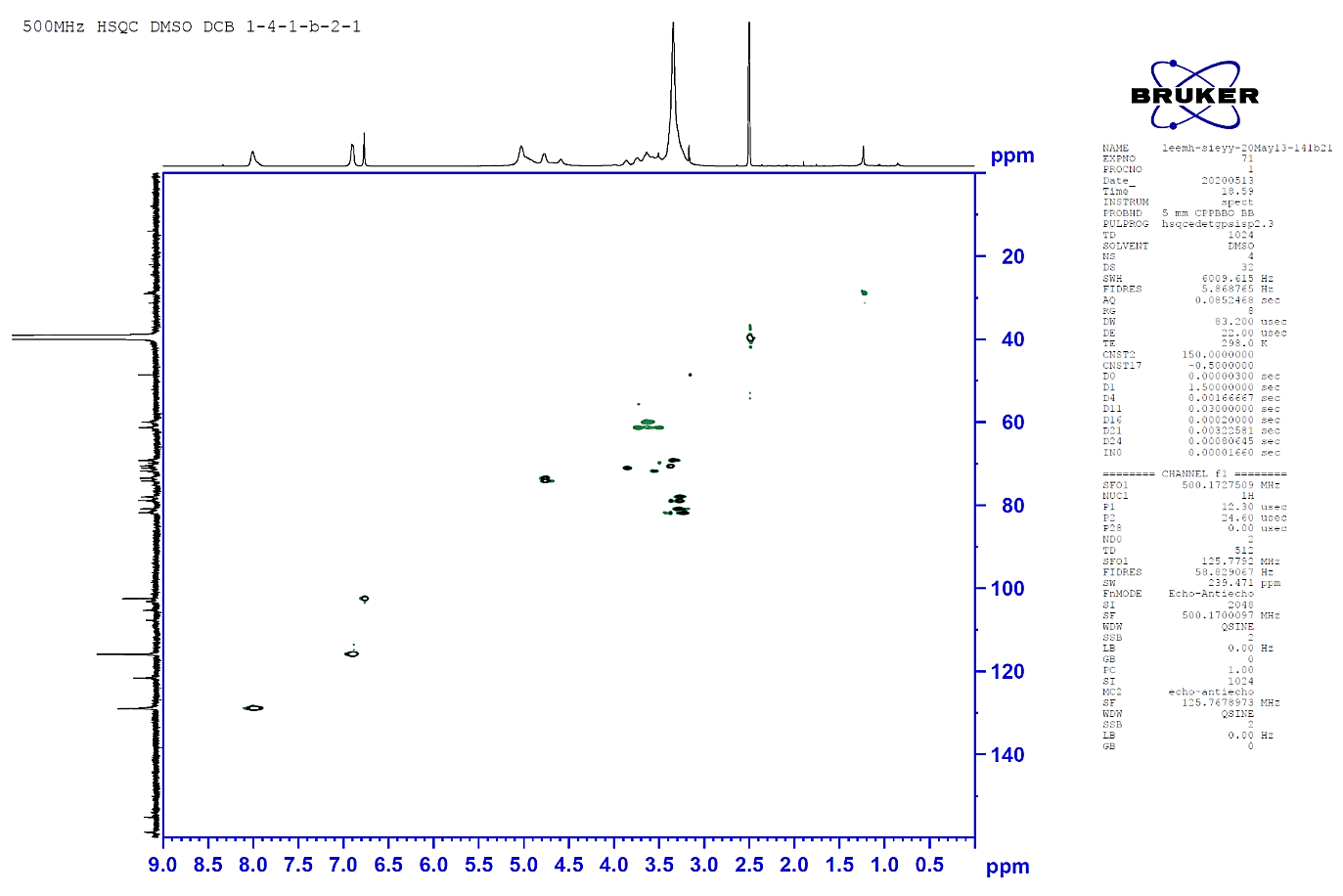


(D)


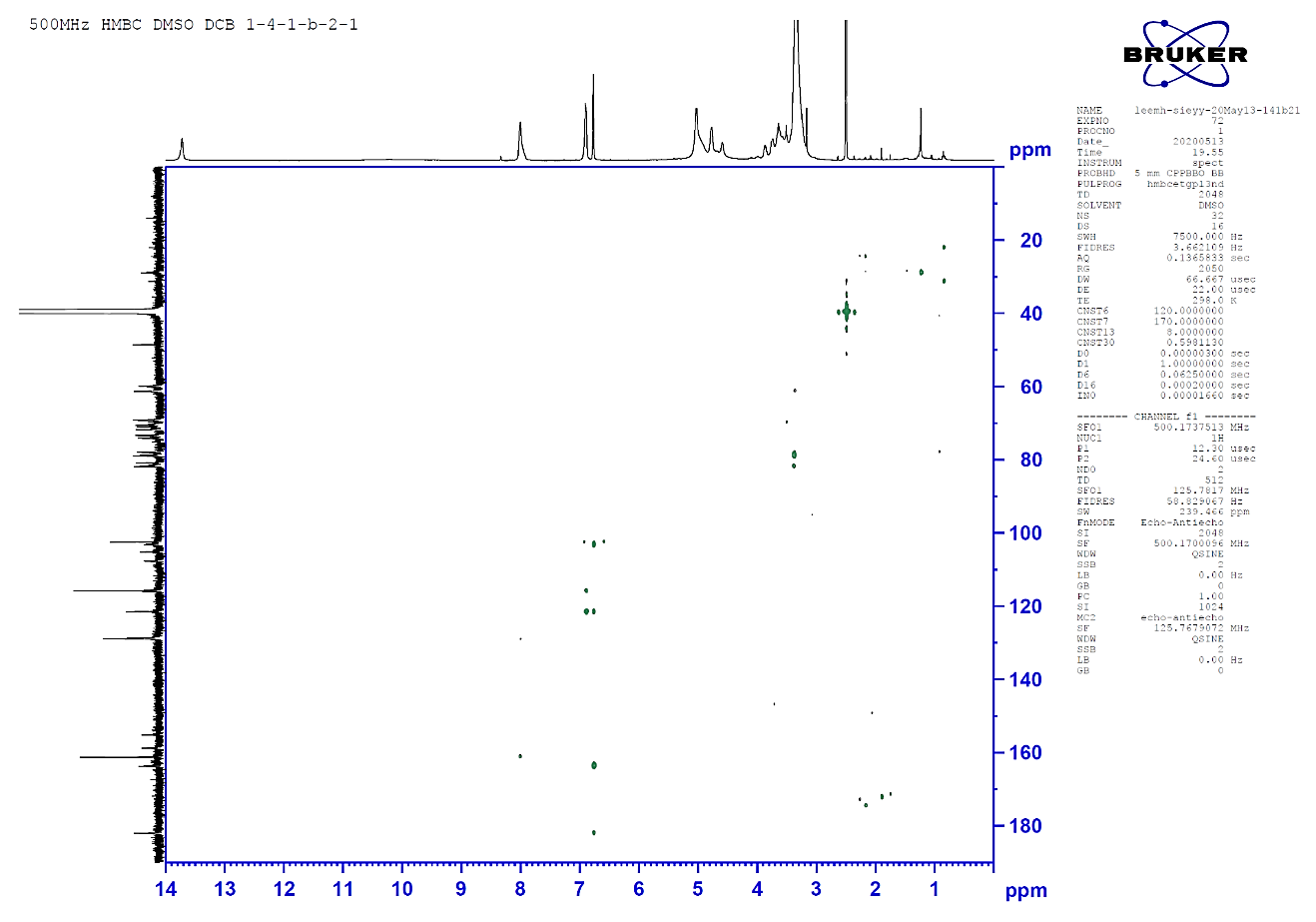


(E)


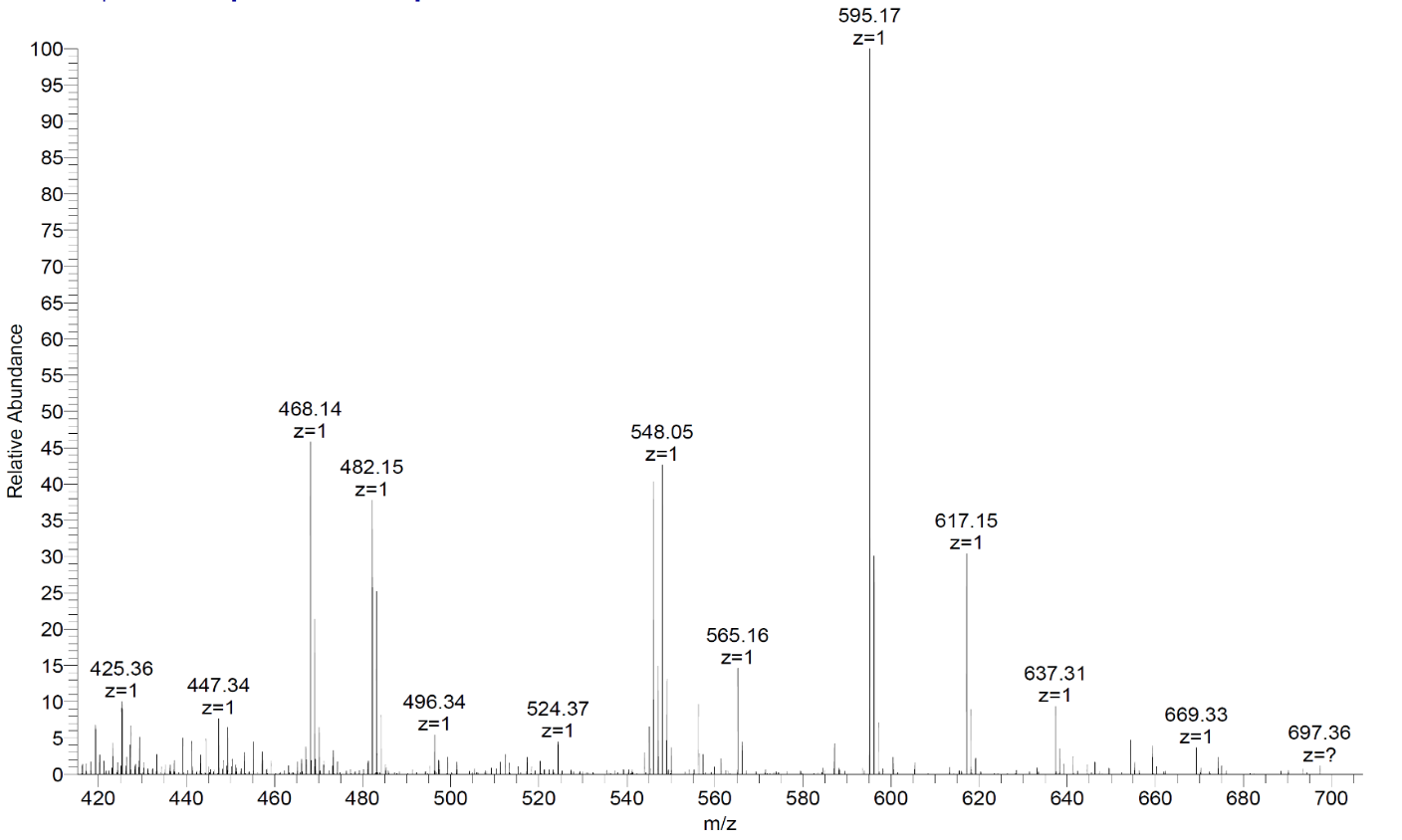


**Figure S2.** Structural identification of compound **2** by (A) ^1^H-NMR, (B) ^13^C-NMR, (C) HSQC-NMR, (D) HMBC-NMR, and (E) high resolution TOF-MS

(A)


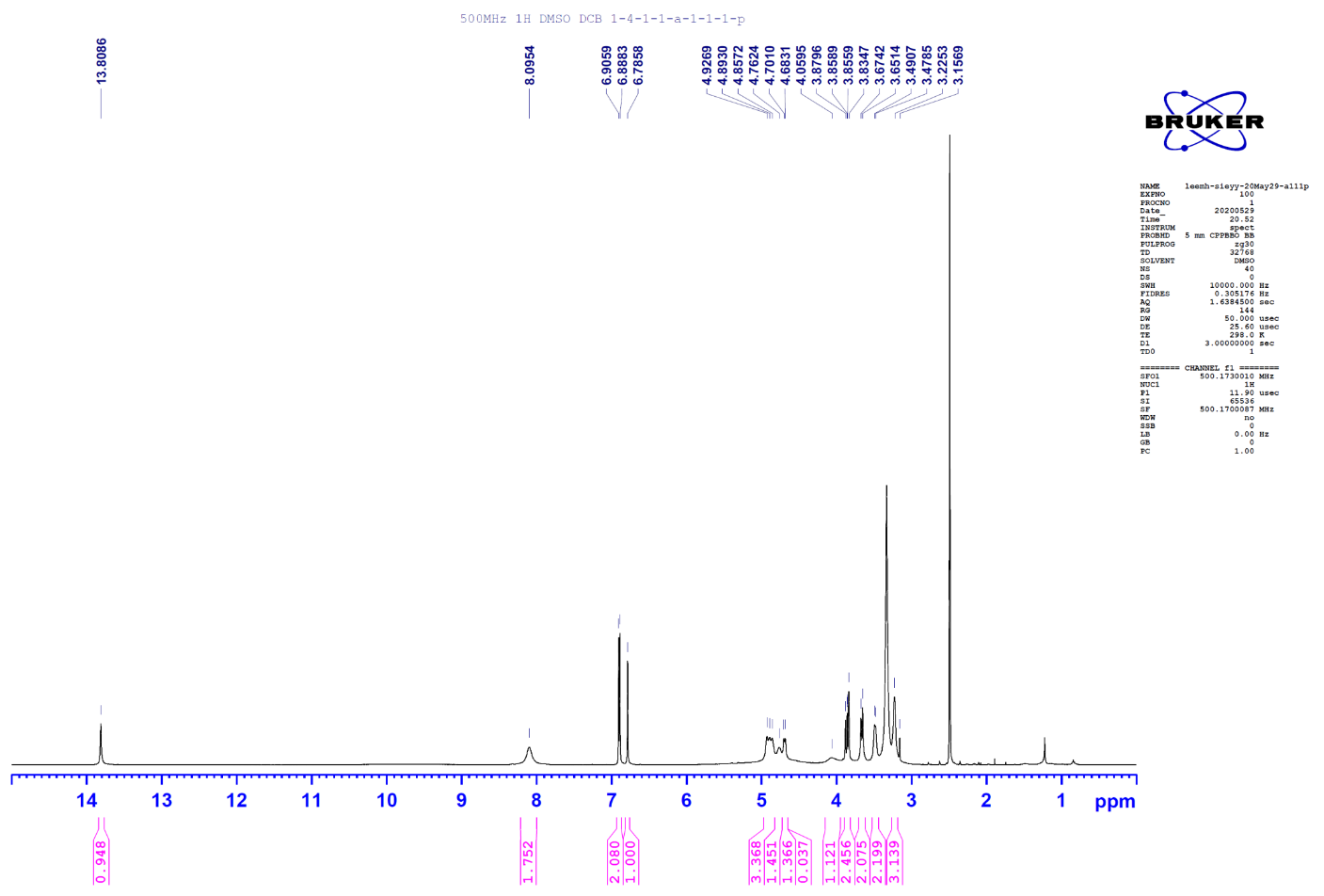


(B)


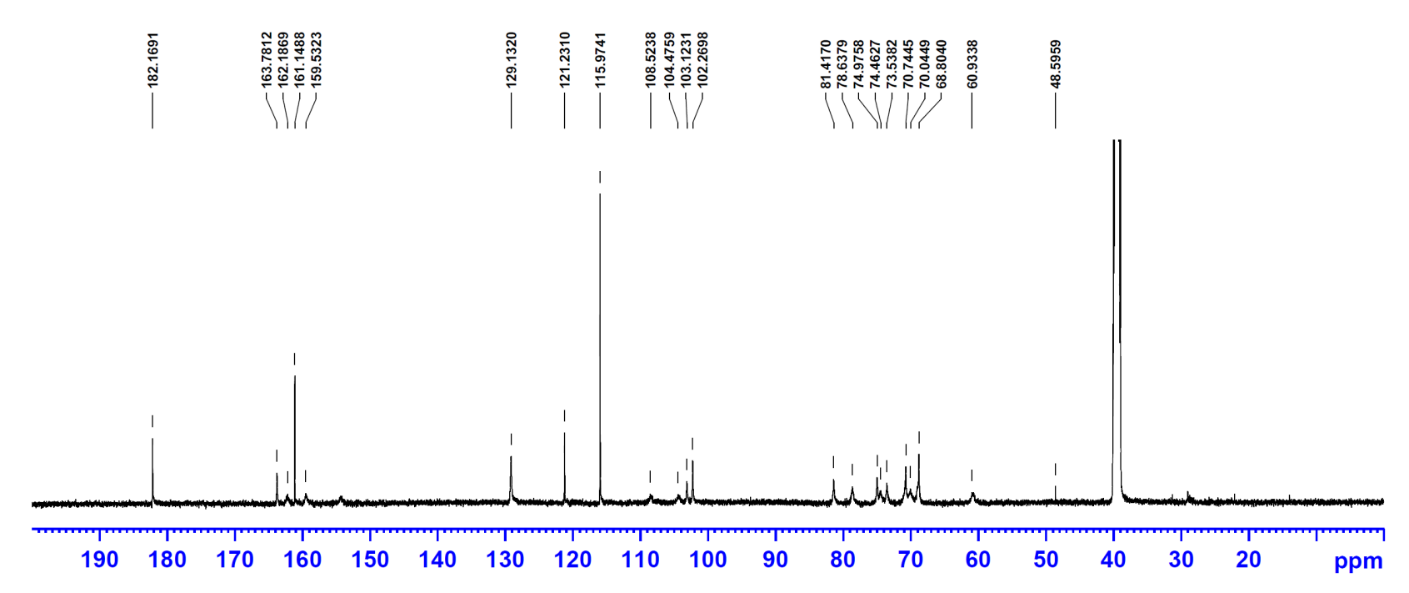


(C)


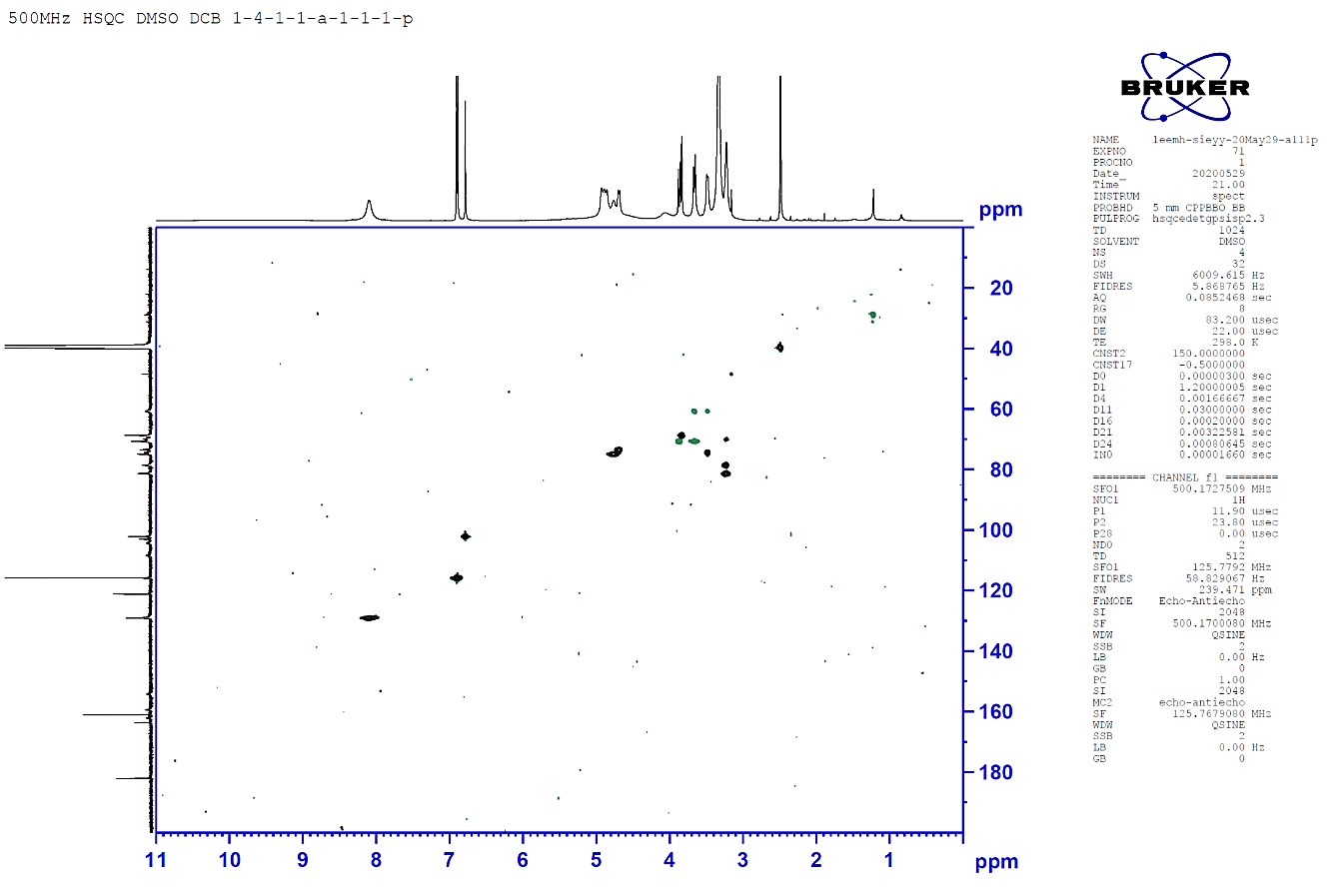


(D)


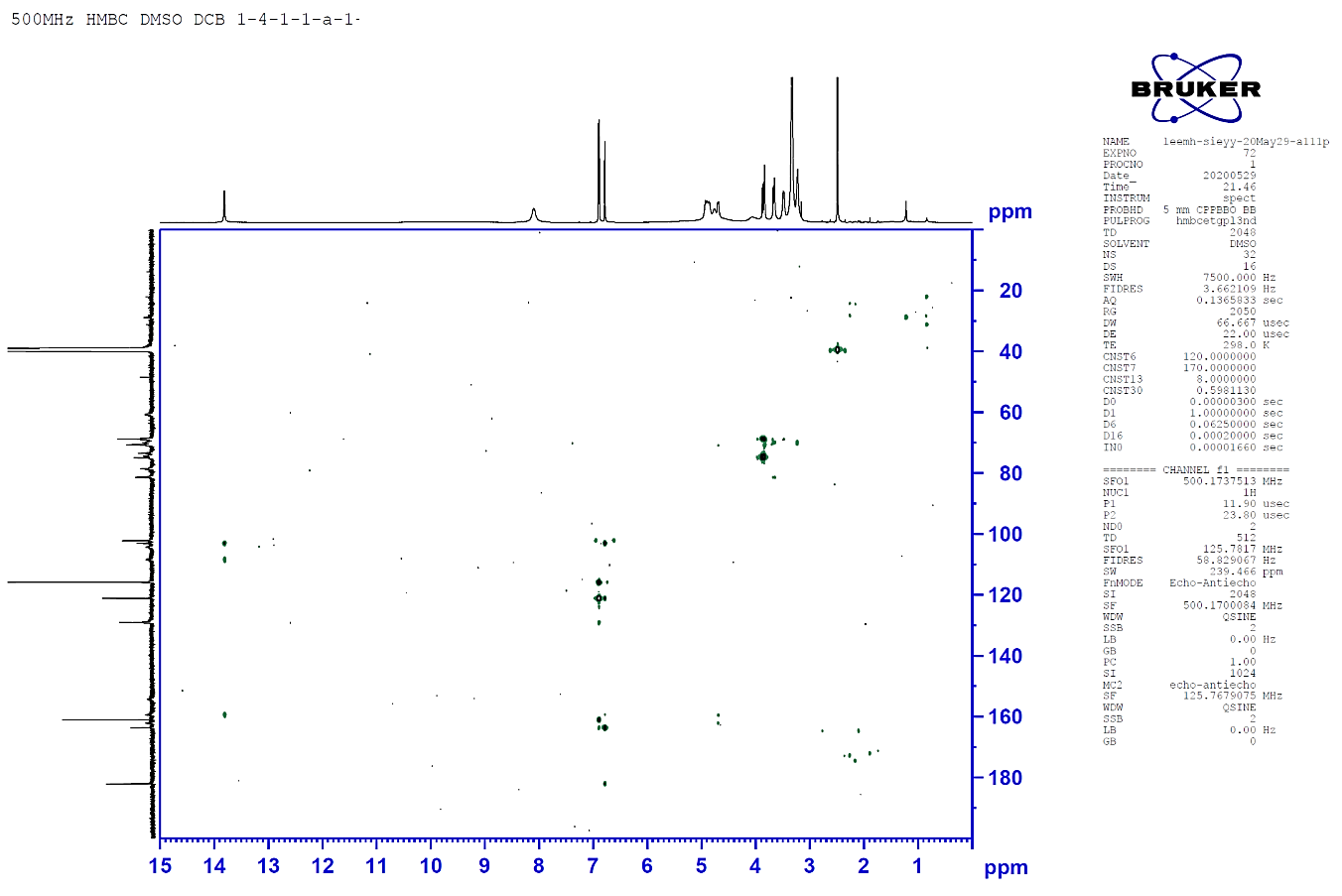


(E)


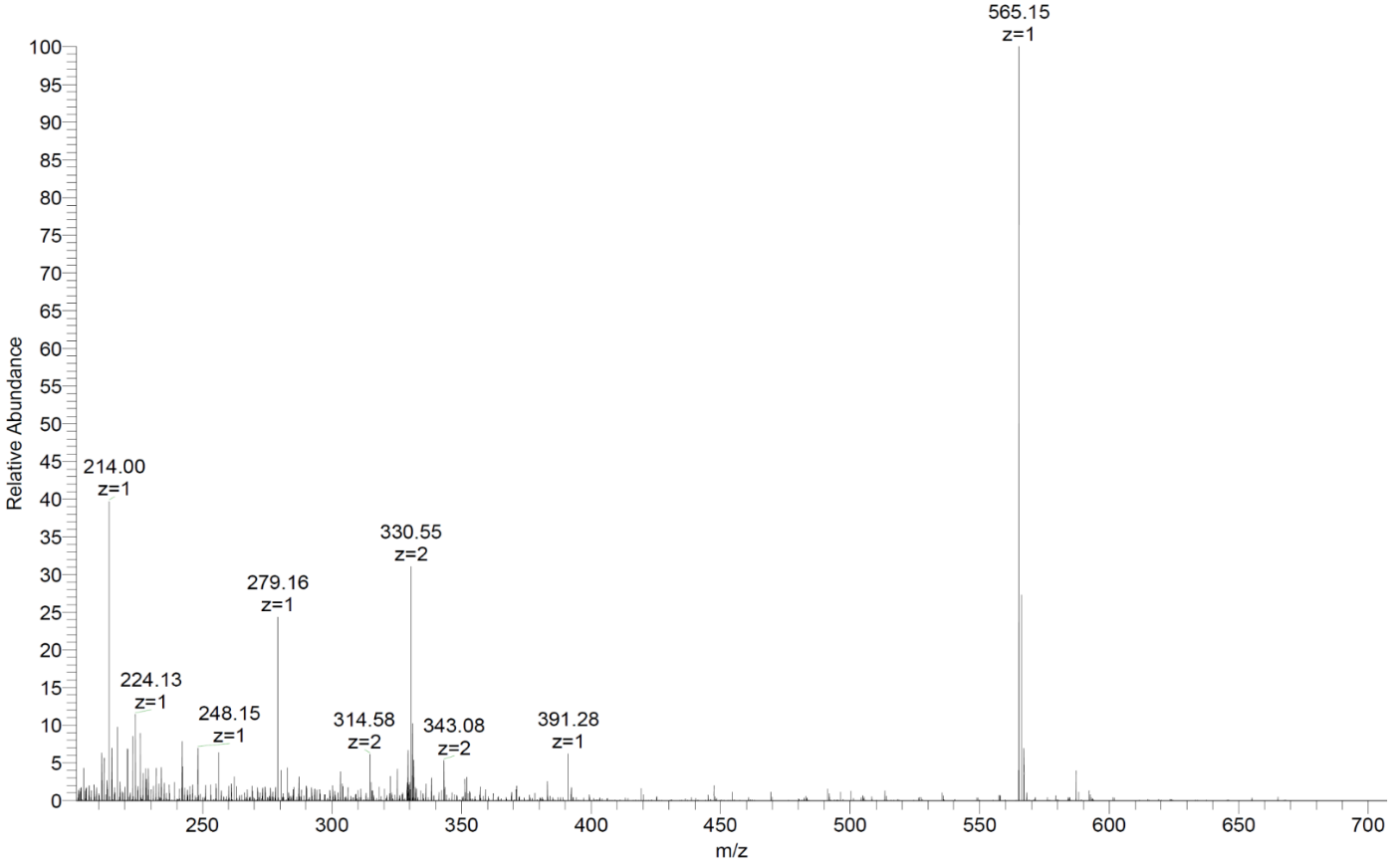


**Figure S3.** (A) The structure of crystal system, (B) average bond length, and (C) summary of crystal data of compound **3**.

(A)


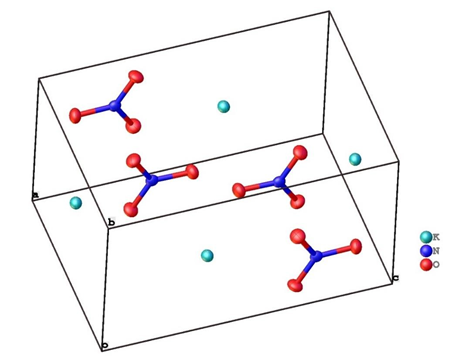


(B)

(C)


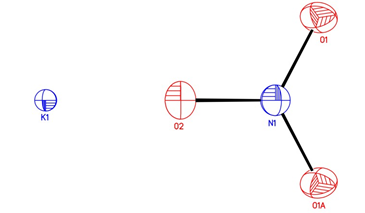


**Figure S4.** The HPLC chromatogram of the overlapping compound **1** and compound **2** with the DC95-EE. The black line, the DC-95EE; the green line, the compound **1** of vicenin II; the purple line, the compound **2** of schaftoside. The analytical Phenomenex Luna PFP(2) (5μm, 250 x 4.6 mm) was performed using Shimadzu SCL-40 chromatography system with an isocratic elution program was set in solvent mixtures of distilled water and acetonitrile as follows: water/acetonitrile containing 0.05% acetic acid, 87/13, at 0 to 30 min; 0/100, at 30.01 to 35 min; 87/13, at 35.01 to 40 min. The concentrations of DC-95EE, compound **1**, and compound **2*,*** respectively, were 100 mg/mL, 1 mg/mL, and 1 mg/mL, and 10 μL was injected for analysis. The flow rate was 1.0 mL/min and the wavelength was set at 335 nm for monitoring. The identified compounds included compound **1** (vicenin II,11.426 min) and compound **2** (schaftoside, 21.341 min).


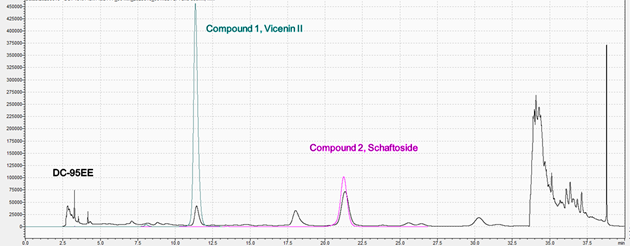

Supplement: Supplementary file 1 — Additional file 1. Fig. S1. Structural identification of compound 1 by A 1H-NMR, B 13C-NMR, C HSQC-NMR, D HMBC-NMR, and E high resolution TOF-MS. Fig. S2 Structural identification of compound 2 by A 1H-NMR, B 13C-NMR, C HSQC-NMR, D HMBC-NMR, and E high resolution TOF-MS. Fig. S3 A The structure of crystal system, B average bond length, and C summary of crystal data of compound 3. Fig. S4 The HPLC chromatogram of the overlapping compound 1 and compound 2 with the DC95-EE. [file 40529_2025_478_MOESM1_ESM.docx]
